# Equatorial thiolate ligand effects in polynuclear dysprosium metallocene single-molecule magnets

**DOI:** 10.1039/d6qi01594h

**Published:** 2026-10-01

**Authors:** Dan Liu, Abdullah A. Al-Kahtani, Liviu Chibotaru, Dinh Cao Huan Do, Fu-Sheng Guo, Richard A. Layfield

**Affiliations:** a School of Science, Changchun Institute of Technology Changchun 130012 P. R. China; b Chemistry Department, College of Science, King Saud University P.O. Box 2455 Riyadh 11451 Saudi Arabia; c Theory of Nanomaterials Group, Department of Chemistry KU Leuven Celestijnenlaan 200F 3001 Leuven Belgium Liviu.Chibotaru@kuleuven.be; d Department of Chemistry, School of Life Sciences, University of Sussex Brighton BN1 9QR UK r.layfield@sussex.ac.uk; e Institute of Fundamental and Frontier Sciences, University of Electronic Science and Technology of China Xiyuan Avenue 2006 Chengdu 611731 P. R. China

## Abstract

Thiolate ligands provide a valuable platform on which to study how soft, polarizable donor atoms affect local magnetic anisotropy and exchange coupling in polynuclear dysprosium single-molecule magnets (SMMs). Here, we report the synthesis of the naphthylthiolate-bridged dysprosium metallocene dimer [(Cp^Me^)_2_Dy(μ-SNaph)]_2_ (Dy_2_S_2_) and trimer [(Cp^Me^)_2_Dy(μ-SNaph)]_3_ (Dy_3_S_3_) (Cp^Me^ = C_5_H_4_Me), obtained selectively through temperature-dependent crystallization from the same reaction mixture. The two compounds enable direct comparison of the influence of nuclearity on magnetic behaviour while retaining closely related dysprosium coordination environments. Both compounds exhibit zero-field SMM behaviour with similar effective barriers to magnetic relaxation of 61 and 64 cm^−1^, respectively, together with significant Raman relaxation at lower temperatures. *Ab initio* calculations reveal strongly axial ground Kramers doublets with comparable crystal field splittings for all Dy^3+^ centres. The calculations accurately reproduce the experimental magnetic susceptibility and isothermal magnetization, revealing that increased nuclearity strengthens the antiferromagnetic exchange without significantly perturbing the local magnetic anisotropy.

## Abstract 2

Temperature-dependent crystallization affords dimeric or trimeric naphthylthiolate-bridged dysprosium metallocenes. Higher nuclearity strengthens antiferromagnetic exchange without significantly perturbing the local magnetic anisotropy.

## Graphical abstract

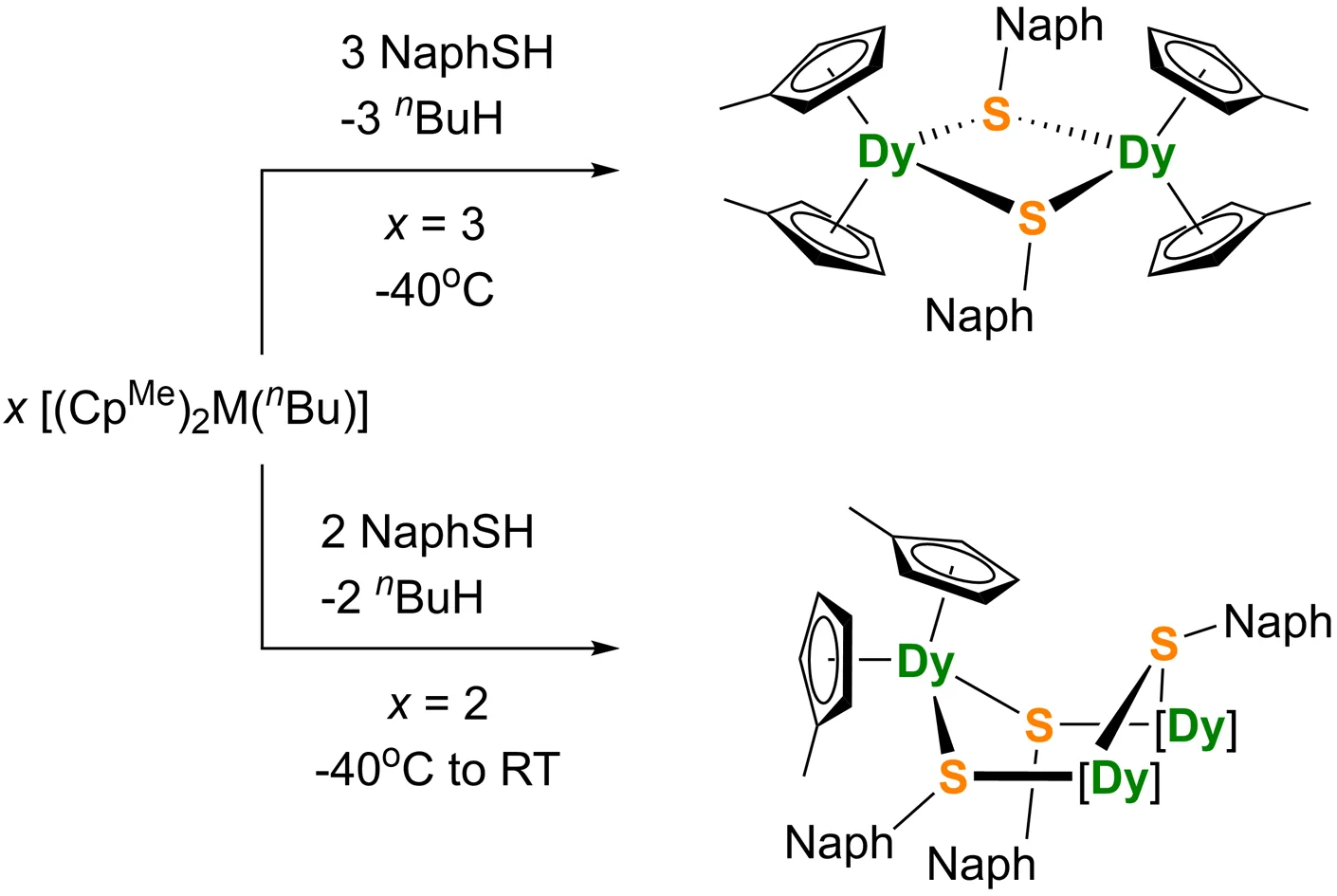

## Introduction

Coordination compounds of f-elements supported by soft-donor ligands attract considerable interest owing to their potential applications in the nuclear sector, particularly the selective separation of lanthanides and actinides from spent fuel.^1,2^ Such separations are generally understood to arise from an apparent “mismatch” between the ionic bonding preferences of hard trivalent lanthanide cations and the greater covalency associated with bonds between early actinides and soft-donor ligands.^3,4^ This mismatch leads to preferential binding to actinides, thereby enabling their extraction.^5^ More broadly, the nature of covalency in f-element compounds continues to motivate fundamental studies of metal–ligand interactions and their influence on chemical reactivity and physical properties, including spectroscopy and magnetism.^6,7^

The role of soft-donor ligands in lanthanide single-molecule magnets (SMMs) is an intriguing topic with considerable scope for development. Whereas most lanthanide SMMs are based on hard O- and/or N-donor ligands,^8–11^ analogues with heavier, softer donor ligands are much rarer.^12–14^ However, several SMMs with heavier donor ligands from groups 14,^15–21^ 15^22–26^ and 16^27–38^ are known. Although lanthanide-ligand interactions are dominated by the electrostatic contribution, the enhanced covalency associated with heavier donor atoms can influence SMM behaviour by modifying charge distribution and enhancing the crystal field strength, both of which impact on magnetic anisotropy. In polynuclear systems, softer ligands also provide a way of influencing SMM properties through magnetic exchange.^11^

Thiolate ligands are especially appealing targets because the relatively diffuse sulphur 3p orbitals may facilitate exchange between lanthanide centres while simultaneously perturbing the crystal field environment at the metal. Polynuclear thiolate-bridged dysprosium compounds therefore represent promising platforms for understanding the interplay between soft-donor coordination, magnetic anisotropy and slow magnetic relaxation. Here, we report the synthesis, structures and magnetic properties of the 1-naphthylthiolate-bridged dysprosium metallocene dimer [(Cp^Me^)_2_Dy(μ-SNaph)]_2_ and the analogous trimer [(Cp^Me^)_2_Dy(μ-SNaph)]_3_ (Cp^Me^ = C_5_H_4_Me, Naph = 1-naphthyl), along with detailed theoretical modelling of their magnetism.

## Results and discussion

### Synthesis and structural properties

The dimer [(Cp^Me^)_2_Dy(μ-SNaph)]_2_ (Dy_2_S_2_) and the trimer [(Cp^Me^)_2_Dy(μ-SNaph)]_3_ (Dy_3_S_3_) were synthesized according to Scheme 1, using the same reaction conditions, in which 1-NaphSH was deprotonated by [(Cp^Me^)_2_Dy(μ-^*n*^Bu)]_2_ ^39^ in diethyl ether at ambient temperature. The two different compounds were obtained by recrystallizing the products at different temperatures. After stirring the reaction for one minute, Dy_2_S_2_ was obtained as colourless crystals in 49% yield following recrystallization at room temperature overnight. In contrast, Dy_3_S_3_ was obtained as yellow plates in 36% yield by storing the nascent reaction solution at −40 °C overnight. The phase purity of the two compounds was established by comparing their powder X-ray diffraction patterns with those calculated using single-crystal X-ray diffraction data, with good agreement in both cases (Fig. S2 and S4).

**Scheme 1:**
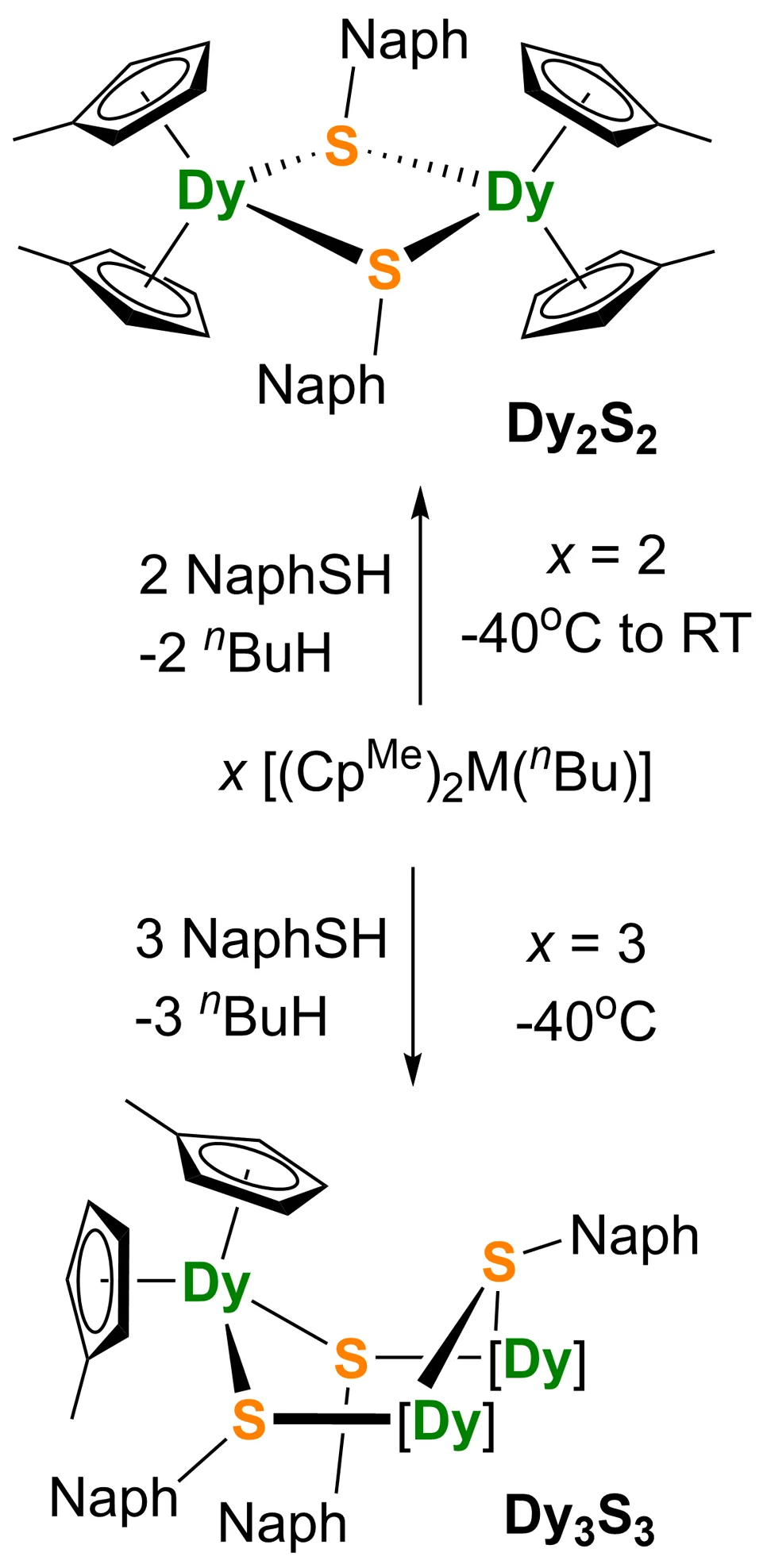
Synthesis of compounds Dy_2_S_2_ and Dy_3_S_3_. [Dy] = Dy(η^5^-C_5_H_4_Me)_2_.

Single-crystal X-ray diffraction reveals that Dy_2_S_2_ crystallizes in the *I*4/*m* space group with a quarter of the centrosymmetric dimer [(Cp^Me^)_2_Dy(μ-SNaph)]_2_ representing the asymmetric unit (Fig. 1 and Tables 1, S1). Each Dy^3+^ ion in the dimer is bound to two η^5^-Cp^Me^ ligands and two sulphur atoms from the [NaphS]^−^ ligands, resulting in approximate *C*_2v_ environments for dysprosium and sulphur. The structure occurs in two disordered forms because the two naphthyl substituents occupy *syn*- and *anti*-positions (50 : 50 occupancy, Fig. 1 and S5). Disorder is also present in the Cp^Me^ ligands, with the methyl groups occupying two distinct positions. Slightly asymmetric coordination of the Cp^Me^ ligands is reflected in a spread of Dy–C distances, which are 2.58(2)–2.63(2) Å. The Dy–Cp^Me^ centroid distances are 2.335(8) Å and 2.349(8) Å, and the Cp^Me^–Dy–Cp^Me^ angles are 125.3(3) and 131.8(3)°. The rhomboidal nature of the central ring in Dy_2_S_2_ is reflected in the Dy–S distances of 2.763(2) and 2.767(2) Å, and the Dy–S–Dy and S–Dy–S angles of 106.92(8)° and 72.62(6)°, respectively. The intramolecular Dy⋯Dy separation in Dy_2_S_2_ is 4.4430(7) Å.

**Fig. 1:**
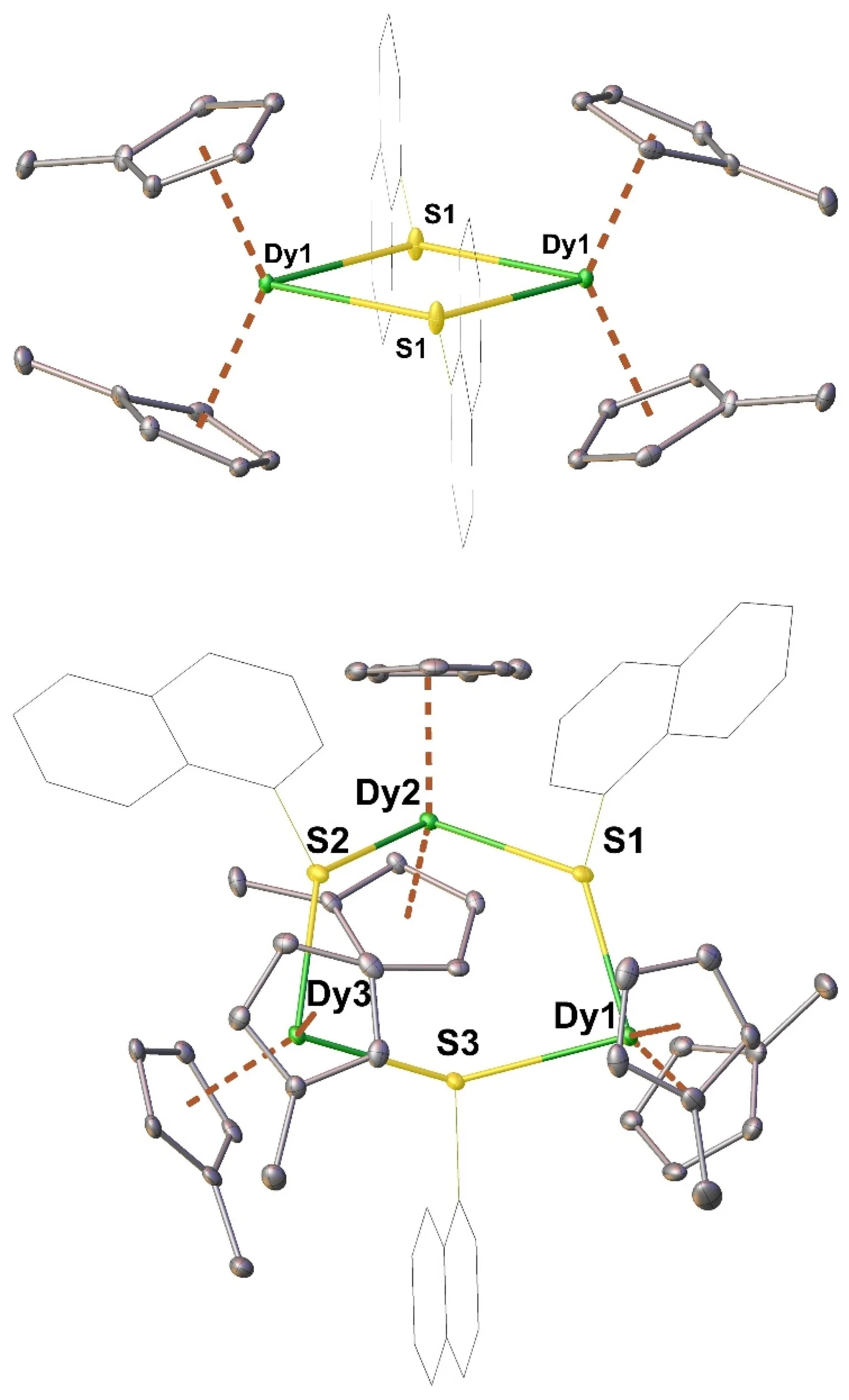
Molecular structures of Dy_2_S_2_ (upper, one disordered form) and Dy_3_S_3_ (lower) (20% thermal ellipsoids). For clarity, hydrogen atoms are not shown, and naphthyl groups are depicted in wireframe. Dy_2_S_2_ is depicted with naphthyl groups in the *anti*-conformation.

**Table 1:** Selected distances (Å) and angles (°) for Dy_2_S_2_ and Dy_3_S_3_

	Dy_2_S_2_	Dy_3_S_3_
Dy–S	2.763(2), 2.767(2)	2.771(3)–2.818(2)
Dy–Cp^Me^(cent)	2.335(8)	2.344(4)–2.363(4)
	2.349(8)	
Dy–C(Cp^Me^)	2.58(2)–2.63(2)	2.581(7)–2.679(8)
Dy⋯Dy	4.4430(7)	5.039(2)–5.293(1)
Dy–S–Dy	106.92(8)	129.08(6)
		130.54(7)
		141.23(7)
S–Dy–S	72.62(6)–73.08(8)	87.16(6)
		88.58(6)
		98.40(6)
Cp^Me^–Dy–Cp^Me^	125.3(3)	126.2(1)
	131.8(3)	126.9(2)
		127.8(1)

Compound Dy_3_S_3_ crystallizes in the monoclinic space group *C*2/*c*, with the full molecule in the asymmetric unit (Fig. 1 and Table S1). The trimer is based on a central six-membered ring with alternating dysprosium and sulphur atoms, resembling a boat-like conformation. The Dy–S bond lengths lie in the range 2.771(3)–2.818(2) Å, with the Dy–S–Dy and S–Dy–S angles being 129.08(6)–141.23(7)° and 87.16(6)–98.40(6)°, respectively. The Dy–C and Dy–Cp^Me^ centroid distances are 2.581(7)–2.679(8) Å and 2.344(4)–2.363(4) Å, respectively, and the Cp^Me^–Dy–Cp^Me^ angles lie in the range 126.18(13)–127.83(13)°. The intramolecular Dy⋯Dy distances in Dy_3_S_3_ are in the range 5.0393(15)–5.2931(11) Å.

Coordination compounds of dysprosium with sulphur-donor ligands are somewhat less numerous than analogous compounds with hard oxygen-donors. Nevertheless, monometallic and multinuclear dysprosium thiolate and sulfide complexes have attracted interest because these softer ligand environments provide an alternative approach to SMMs based on the hard–soft mismatch between metal and ligand. Examples include the diethyldithiocarbamate-ligated monometallic SMM [DyL^ON3^(Et_2_NCS_2_)_2_]·0.5THF with a high effective barrier of 443 cm^−1^,^32^ the thiolate-bridged dimer [(Cp^Me^)_2_Dy(μ-SSiPh_3_)]_2_ with a barrier of 133 cm^−1^,^28^ and tetrametallic SMMs [Ln_4_{N(SiMe_3_)_2_}_4_(μ-SEt)_8_(μ_4_-SEt)]^−^ (Ln = Tb, Dy).^30^ More recently, the dimetallic sulfide-bridged complexes [{(Cp*)_2_Dy(THF)}_2_(μ-S)] and its solvent-free analogue [{(Cp*)_2_Dy}_2_(μ-S)] (Cp* = C_5_Me_5_) were reported to show solvent-dependent field-induced slow magnetic relaxation,^27^ and isomers of the cluster fullerene Dy_2_S@C_82_ exhibit SMM properties in zero DC field.^31^

### Magnetic properties

The temperature dependence of the molar magnetic susceptibility of Dy_2_S_2_ and Dy_3_S_3_ was measured in an applied field of 10 kOe in the temperature range 2–300 K. The *χ*_M_*T* product at 300 K for Dy_2_S_2_ is 27.99 cm^3^ K mol^−1^, in good agreement with the expected value of 28.34 cm^3^ K mol^−1^ for two isolated Dy^3+^ ions (*S* = 5/2, *L* = 5, ^6^H_15/2_, *g* = 4/3; *C* = 14.17 cm^3^ K mol^−1^) (Fig. S6).^40^ On lowering the temperature, *χ*_M_*T* decreases gradually down to about 50 K, and then more rapidly to reach 8.16 cm^3^ K mol^−1^ at 2.0 K. Compound Dy_3_S_3_ shows similar behaviour, with a *χ*_M_*T* value of 41.76 cm^3^ K mol^−1^ at 300 K, and 13.72 cm^3^ K mol^−1^ at 2 K (Fig. S7). The thermal dependence of the susceptibility for both compounds can be attributed to a combination of thermal depopulation of the excited crystal field levels within the ^6^H_15/2_ ground multiplet of each Dy^3+^, along with the effect of antiferromagnetic exchange and, possibly, magnetic blocking.^41^ The field-dependence of the magnetization (*M*) for Dy_2_S_2_ and Dy_3_S_3_ at 1.8 K is also consistent with their di- and tri-metallic nature, respectively, with *M* reaching 10.56*Nβ* and 14.64*Nβ*, respectively at 7 T (Fig. S6 and S7).

Looking to establish SMM behaviour in Dy_2_S_2_ and Dy_3_S_3_, the real (*χ*′) and imaginary (*χ*″) components of the AC susceptibility were measured in an oscillating field of 1.55 Oe and zero DC field, with AC frequencies in the range *ν* = 2–1400 Hz (Fig. 2 and S8, S11). For Dy_2_S_2_, frequency-dependent maxima were observed in the *χ*″(*ν*) plots in the temperature range 6–20 K, and for Dy_3_S_3_ maxima were observed in the range 8–26 K. Relaxation times (*τ*) were extracted by fitting the semi-circular Cole–Cole plots of *χ*″(*χ*′) (Fig. S9 and S12), using *α*-parameters of 0.13–0.19 for Dy_2_S_2_ (Table S2) and 0.15–0.50 for Dy_3_S_3_ (Table S3), indicating a narrow distribution of relaxation times for the dimer and a much broader distribution for the trimer.

**Fig. 2:**
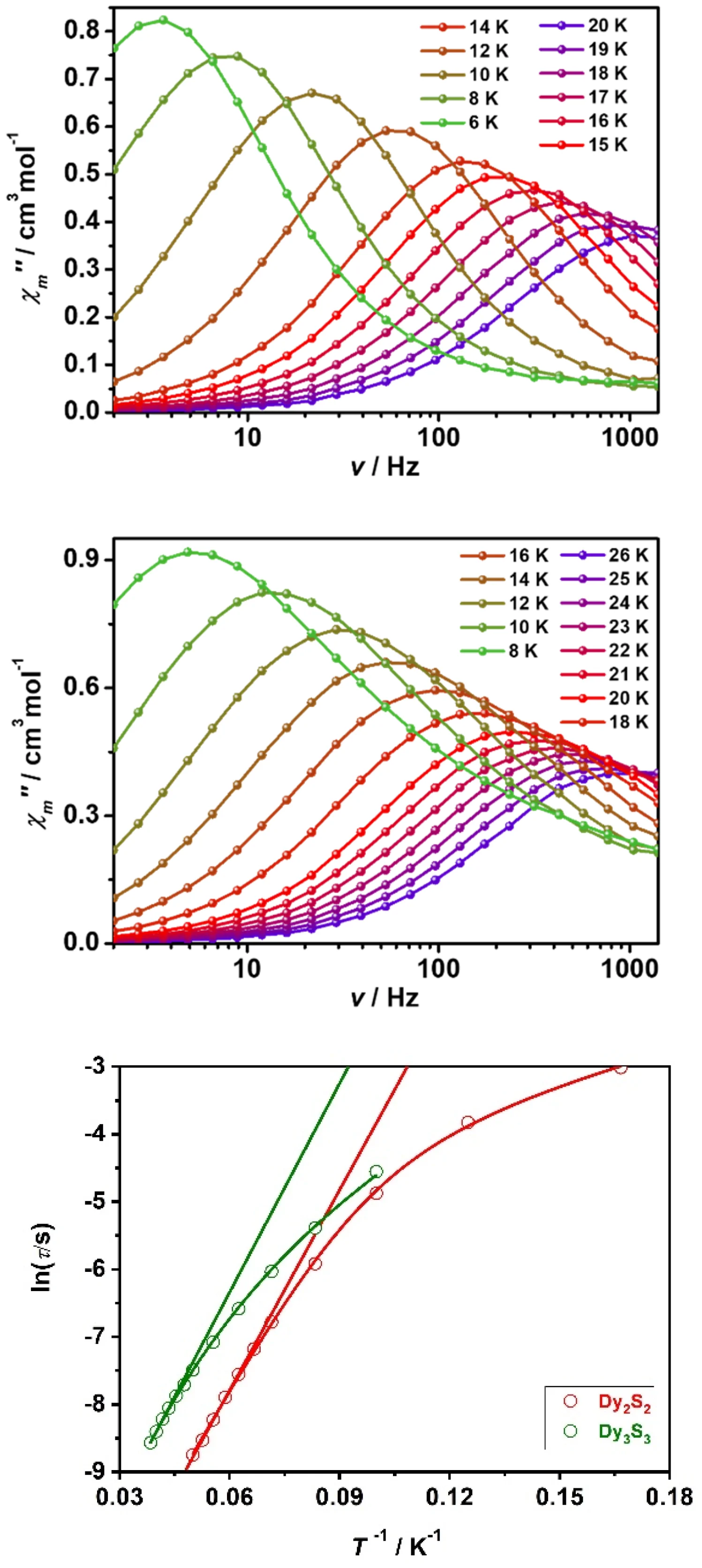
Plots of *χ*″(*ν*) for Dy_2_S_2_ (upper) and Dy_3_S_3_ (middle), and plots of ln *τ vs. T*^−1^ (lower) in zero applied field. Solid lines represent best fits of the data using the parameters stated in the SI.

Plotting ln(*τ*) as a function of *T*^−1^ for Dy_2_S_2_ revealed a linear dependence in the region 15–20 K indicative of Orbach relaxation (Fig. S10). Curvature in the plot at lower temperatures suggests a shift towards dominant Raman relaxation, whereas a temperature-independent regime was not observed in the temperature window of the measurement. A good fit of the ln(*τ*) *vs. T*^−1^ data was obtained using *τ*^−1^ = *τ*_0_^−1^*e*^−*U*_eff_/*k*_^B^_*T*^ + *CT*^*n*^, where *τ*_0_ denotes the attempt time, *U*_eff_ is the effective energy barrier, and *C* and *n* are the Raman coefficient and Raman exponent, respectively. For Dy_2_S_2_, this analysis produced *τ*_0_ = 1.32 × 10^−6^ s, *U*_eff_ = 61(2) cm^−1^, *C* = 0.127 s^−1^ K^−*n*^, and *n* = 2.82. The same analysis for Dy_3_S_3_ revealed a linear regime at 23–26 K and curvature in the data at lower temperatures, again without a temperature-independent regime (Fig. S13). Analysis of the relaxation time data produced *τ*_0_ = 3.95 × 10^−5^ s, *U*_eff_ = 64 cm^−1^, *C* = 0.01 s^−1^ K^−*n*^, and *n* = 4.05.

The effective barriers for both compounds are similar, with the *τ*_0_ values consistent with relaxation *via* small effective barriers. Indeed, the large *τ*_0_ values for both compounds indicate that their Orbach processes are not pure within the measured temperature range, and that Raman processes have already begun to dominate. The true energy barriers for each compound should therefore be higher than those obtained from the data fits. Both compounds exhibit low Raman exponents relative to the ideal value of *n* = 9 for a Kramers ion, consistent with the increasingly common observation that Raman relaxation in dysprosium metallocene SMMs is strongly influenced by low-energy molecular and lattice vibrations.^42,43^ The lower *n* and larger *C* value for Dy_2_S_2_ indicate more efficient low-temperature Raman relaxation than in Dy_3_S_3_, perhaps due to stronger coupling to soft Dy–S vibrational modes. However, because the fits cover a limited temperature range, these parameters should not be over-interpreted.

Magnetic hysteresis measurements on Dy_2_S_2_ at 1.8 K produced no open loops (Fig. S14). The measurements employed variable field-sweep rates of 0.4–2.5 mT s^−1^, with an average rate of 1.1 mT s^−1^ and the slowest rate applied near zero field. For Dy_3_S_3_, a small separation between the forward and reverse sweeps was observed at 1.8 K over approximately |*H*| = 0.3–0.9 T, whereas the loop was closed at 2.2 K (Fig. S15). Neither compound showed appreciable coercivity or remanent magnetization. Given the relatively slow sweep rate around zero field, the effectively closed loops show that magnetic relaxation is rapid on the timescale of the experiment. This behaviour is consistent with the continued operation of the Raman process identified from the AC susceptibility data. No distinct zero-field step attributable to QTM was observed, although the absence of such a feature does not exclude QTM. Consistent with this interpretation, a QTM term was not required to fit the relaxation-time data.

### Theoretical studies

*Ab initio* calculations of each compound were carried out using the CASSCF/RASSI-SO/SINGLE_ANISO approach implemented in the MOLCAS 8.4 program package.^44^ Two basis sets were employed (see Table S4 and SI for full details). In order to include the effect of spin–orbit coupling (SOC) on the dysprosium centres, CASSCF calculations were performed at the ground sextet multiplet (*S* = 5/2) with all 21 configurations, the first excited quartet multiplet (*S* = 3/2) with all 224 configurations and the secondexcited doublet multiplet (*S* = 1/2) with all 490 configurations. After the CASSCF calculations, the RASSI calculation of RAS state interaction was performed by including 21 configurations for the ground spin (*S* = 6), 128 configurations for the first excited spin (*S* = 4) and 130 configurations for the second excited spin (*S* = 2). The coordinates for the calculations were taken from the crystallographic structures, while the neighbouring Dy^3+^ ions were replaced by Lu^3+^. Only the hydrogen atom positions were optimized using the UDFT D3BJ calculation with the BP functional and SVP basis set using the ORCA program.^45^

The calculated *g*-tensors of the lowest-lying Kramers doublets (KDs) of the Dy^3+^ centres in Dy_2_S_2_ and Dy_3_S_3_ approach the Ising limit, with *g*_*x*_ and *g*_*y*_ in the range 0.000–0.003 and *g*_*z*_ being 19.28–19.48 under basis set 2 (Table 2). These results show that the magnetic ground state of each Dy^3+^ has dominant |*M*_*J*_| = 15/2 character (approximately 90%), with the cyclopentadienyl ligands providing a strong axial crystal field, albeit with non-negligible (7–10%) |*M*_*J*_| = 11/2 character (Tables S5–S8). In the case of Dy_2_S_2_, the first-excited KD in the two disordered forms is also highly axial with roughly 94% |*M*_*J*_| = 13/2 character, with the calculated energy spectrum revealing that this KD lies 119 cm^−1^ above the ground state (Table 3). The magnetic axes in the ground KD are oriented towards the cyclopentadienyl rings and are disposed at angles of 83.5° and 63.0–64.1° with respect to the {Dy_2_S_2_} and {Dy_3_S_3_} mean planes, respectively. The axes in the corresponding first- and second-excited KDs are essentially co-linear, with calculated deviations of 7.6° and 3.1°, respectively, from the ground KD (Fig. 3 and Table S9). An appreciable transverse component of the crystal field in Dy_2_S_2_ is apparent in the third-excited KD, which lies 387 cm^−1^ above the ground KD, with the magnetic axis lying at an angle of 32.6° relative to the ground KD.

**Fig. 3:**
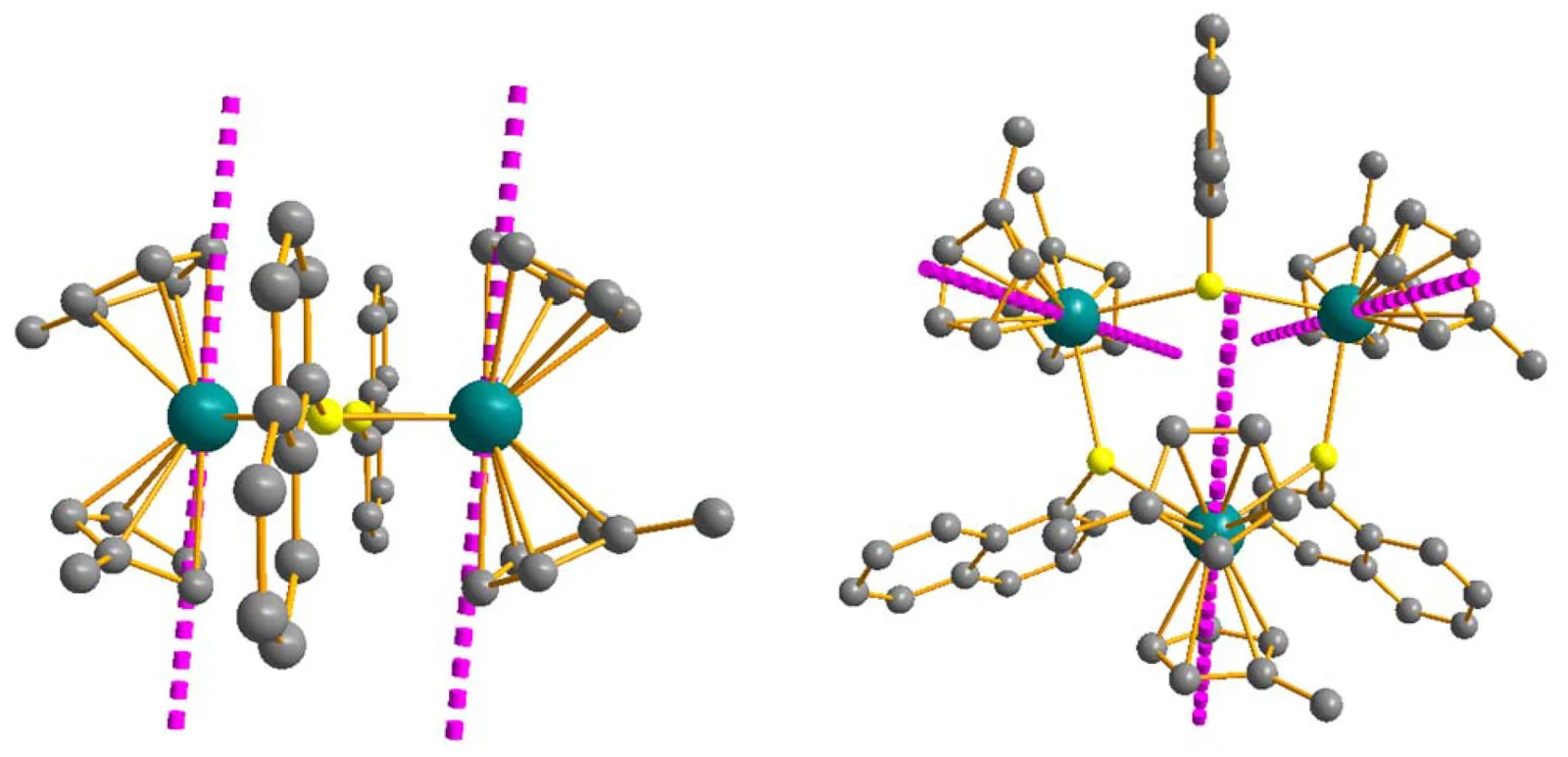
Easy axes of magnetization in the ground KDs of Dy_2_S_2_ (left) and Dy_3_S_3_. Dy = teal, S = yellow, C = grey. Hydrogen atoms are not shown.

**Table 2:** The *g*-factors of the four lowest KDs of Dy^3+^ centres in Dy_2_S_2_ and Dy_3_S_3_, calculated using basis set 2

KD		Dy_2_S_2_	Dy_3_S_3_ (Dy1)	Dy_3_S_3_ (Dy2)	Dy_3_S_3_ (Dy3)
1	*g*_*x*_	0.001	0.000	0.000	0.000
	*g*_*y*_	0.003	0.001	0.001	0.000
	*g*_*z*_	19.275	19.318	19.480	19.466
2	*g*_*x*_	0.006	0.000	0.002	0.001
	*g*_*y*_	0.009	0.001	0.003	0.001
	*g*_*z*_	17.033	16.961	17.132	17.110
3	*g*_*x*_	0.084	0.010	0.085	0.037
	*g*_*y*_	0.113	0.077	0.117	0.041
	*g*_*z*_	14.890	14.869	14.984	14.904
4	*g*_*x*_	1.255	0.005	2.057	1.661
	*g*_*y*_	3.644	0.156	2.695	3.846
	*g*_*z*_	10.581	19.399	10.239	13.166

**Table 3:** Energies (cm^−1^) of the eight KDs within the ground multiplet of the Dy^3+^ ions in Dy_2_S_2_ and Dy_3_S_3_, calculated using basis set 2

KD	Dy_2_S_2_	Dy_3_S_3_ (Dy1)	Dy_3_S_3_ (Dy2)	Dy_3_S_3_ (Dy3)
1	0.0	0.0	0.0	0.0
2	118.5	127.3	134.1	133.0
3	296.0	293.0	267.4	308.5
4	386.9	324.3	319.5	396.2
5	405.7	370.0	350.7	416.3
6	452.5	405.3	390.1	466.6
7	486.4	457.4	408.2	503.6
8	669.8	621.6	466.9	658.1

The energy spectrum calculated for Dy_3_S_3_ features first-excited KDs for the three distinct Dy^3+^ ions at 127–134 cm^−1^ above the ground KD and oriented at angles of 5.1–8.6° (Tables 3 and S9). The *g*-tensors of the second excited KDs for Dy_3_S_3_ feature appreciable transverse components despite their main magnetic axes adopting similar orientations (3.8–12.1°) relative to the ground KDs. The highly axial magnetic ground states calculated for the individual Dy^3+^ ions in Dy_2_S_2_ and Dy_3_S_3_ are broadly in agreement with their observed SMM properties. Indeed, according to *ab initio* results, the SMM behaviour in both compounds is naturally explained as arising due to the blocking of QTM in individual Dy^3+^ centers.^46–49^ However, we note that the calculated relaxation barriers for the two compounds appear to be somewhat higher than those observed experimentally (Fig. S16 and S17). This is likely to reflect the fact that the experimentally determined *U*_eff_ values represent effective kinetic barriers, with magnetic relaxation proceeding through more efficient under-barrier pathways rather than *via* thermal population of the higher-lying crystal field states.

Further account of the exchange coupling between dysprosium sites is expected to enhance magnetic blocking at low temperature. The exchange multiplets in complexes with strongly axial sites are well described within the Lines approximation. The total magnetic interaction between dysprosium ions was determined as a sum of dipolar and conventional exchange interactions. After projecting this interaction onto the space of the ground Kramers doublets on the dysprosium sites, and considering their strong axiality, the Hamiltonian of the total magnetic interaction acquires the Ising form expressed in terms of local pseudospins *S̃*_Dy_ corresponding to the ground KD on each site (*S̃*_Dy_ = 1/2). The following equations describe the interactions for Dy_2_S_2_ and Dy_3_S_3_, respectively. The terms *J*^dip^ and *J*^exch^ denote the dipolar and exchange contributions, respectively, to the magnetic coupling between neighbouring Dy^3+^ centres. The total magnetic interaction is therefore expressed as the sum of dipolar and exchange contributions within the Ising approximation, with *J*^dip^_*i*–*j*_ and *J*^exch^_*i*–*j*_ describing the pairwise interactions between Dy^3+^ centres *i* and *j*. Note that the pseudospin projection on the anisotropy axis (*Z*) corresponds to the same quantization axis on all sites due to the symmetry of the complexes.*Ĥ* = −(*J*_dip_ + *J*_exch_)*S̃*_Dy_1_*Z*_*S̃*_Dy_2_*Z*_
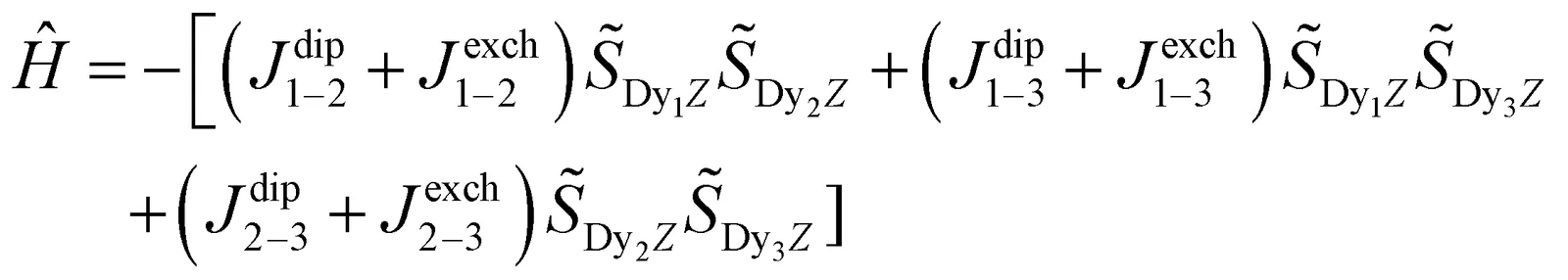


In order to evaluate the Lines exchange parameters, broken symmetry density functional theory (BS-DFT) calculations were carried out at the B3LYP/SVP level with the ORCA program.^50^ The Dy^3+^ ions were replaced by Gd^3+^, while the coordinates of other atoms were kept at their initial positions as in the experimentally determined structures. The exchange coupling parameters were derived by employing the generalized algorithm for calculation of Heisenberg exchange constants in multi-spin systems.^51^ The ground spin of Gd^3+^ is 7/2, and of Dy^3+^ is 5/2. The *J*(Dy–Dy) was therefore determined by multiplying the *J*(Gd–Gd) parameter by a factor of 49/25.^46^
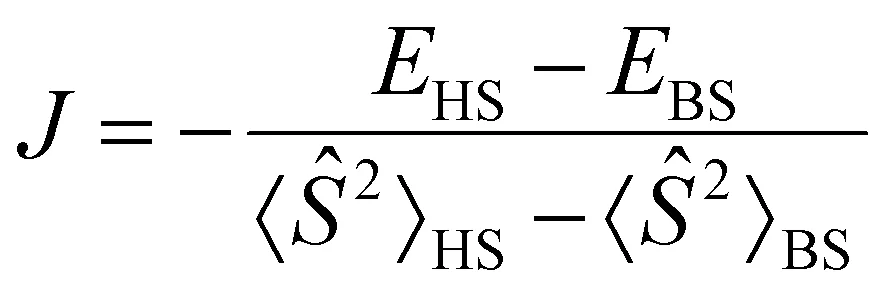


The dipolar coupling parameters were extracted directly from the *ab initio* results (Table 4). By slightly varying the value of the obtained exchange parameters, we were able to calculate the temperature-dependent magnetic susceptibility and the field-dependent magnetization of both complexes in good agreement with the experimental data (Fig. S18–S21). From Table 4, we can see that the dipolar interaction is smaller than the exchange interaction in both compounds, and the overall magnetic exchange interaction between Dy^3+^ centres is antiferromagnetic. The smaller dipolar interaction compared to the exchange interaction can be explained by the ability of soft sulphur donor atoms to promote relatively strong exchange, as has been observed in some related S-bridged transition metal compounds.^52^ The smaller dipolar interaction in Dy_3_S_3_ is consistent with the longer Dy⋯Dy separations in the trimer, which exceed those in Dy_2_S_2_ by 0.70 Å on average. The overall antiferromagnetic exchange coupling is characteristically weak for polymetallic lanthanide compounds, and here it is apparent that the coupling in Dy_3_S_3_ is generally stronger than that in Dy_2_S_2_.

**Table 4:** Magnetic Ising exchange interaction (cm^−1^) in Dy_2_S_2_ and Dy_3_S_3_

	*J*_DFT_	*J*_dip_	*J*_exch_
Dy_2_S_2_	−1.96	−1.64	−4.054
Dy_3_S_3_	−8.65	−0.79	−8.119
	−5.57	−0.76	−5.567
	−2.50	−1.08	−2.503

The strong enhancement of magnetization blocking by inter-site magnetic coupling predominantly of the exchange type is the reason why the corresponding signal is not present in the low-frequency domain of out-of-phase AC magnetic susceptibility. As such, the observed magnetization dynamics are consistent with intra-site magnetic relaxation of the Dy^3+^ sites.

## Conclusions

In summary, deprotonation of 1-naphthylthiol with [(Cp^Me^)_2_Dy(μ-^*n*^Bu)]_2_ produced dimeric [(Cp^Me^)_2_Dy(μ-SNaph)]_2_ (Dy_2_S_2_) and trimeric [(Cp^Me^)_2_Dy(μ-SNaph)]_3_ (Dy_3_S_3_). The different aggregation states of the two complexes highlight a remarkable sensitivity to the crystallization temperature, providing a rare opportunity to study the influence of cluster nuclearity and magnetic exchange on the SMM properties while keeping the basic magnetic building block constant.

Both thiolate-bridged complexes were found to show SMM behaviour in zero applied field, with remarkably similar barriers of 61 cm^−1^ and 64 cm^−1^, respectively. Analysis of the magnetic relaxation dynamics reveals a significant contribution from Raman relaxation at lower temperatures. *Ab initio* calculations show strongly axial ground Kramers doublets for all Dy^3+^ ions together with very similar crystal field splittings, and they accurately reproduce the experimental magnetic susceptibility and magnetization data. The calculations further demonstrate that increasing nuclearity enhances antiferromagnetic exchange between the Dy^3+^ centres without significantly perturbing the local crystal field anisotropy. More broadly, these results demonstrate that structurally flexible naphthylthiolate ligands provide an effective strategy for introducing sulphur-based magnetic exchange into highly axial dysprosium metallocenes, offering a promising platform for the rational design of exchange-coupled lanthanide SMMs with soft donors.

## Author contributions

Abdullah A. Al-Kahtani: formal analysis, investigation, methodology, writing – review and editing. Liviu Chibotaru: conceptualization, funding acquisition, project administration, supervision, writing – original draft, writing – review and editing. Dinh Cao Huan Do: formal analysis, writing – review and editing. Fu-Sheng Guo: formal analysis, investigation, methodology, writing – review and editing. Richard A. Layfield: conceptualization, funding acquisition, project administration, supervision, writing – original draft, writing – review and editing. Dan Liu: formal analysis, investigation, methodology, writing – review and editing.

## Conflicts of interest

There are no conflicts to declare.

## Supplementary Material

QI-013-D6QI01594H-s001

## Data Availability

Additional research data supporting this publication are available at https://doi.org/10.25377/sussex.32948534. Supplementary information (SI) is available. See DOI: https://doi.org/10.1039/d6qi01594h. CCDC 2572223 and 2572224 contain the supplementary crystallographic data for this paper.^53*a*,*b*^
