# Supplementary material for: Equatorial thiolate ligand effects in polynuclear dysprosium metallocene single-molecule magnets
Source: Inorg Chem Front. 2026 Oct 1. Online ahead of print. doi: 10.1039/d6qi01594h (PMC13644314; doi:10.1039/d6qi01594h)
Supplement: Supplementary file 1 [file QI-OLF-D6QI01594H-s001.pdf]

Supplementary Information (SI) for Inorganic Chemistry Frontiers.
This journal is © the Partner Organisations

Supporting Information

Equatorial Thiolate Ligand Effects in Polynuclear Dysprosium Metallocene Single-Molecule Magnets

Dan Liu,[a] Abdullah A. Al-Kahtani,[b] Liviu Chibotaru,*[,c] Dinh Cao Huan Do,[d] Fu-Sheng Guo,[e] Richard A. Layfield,*[,a]

[a] School of Science, Changchun Institute of Technology, Changchun 130012, P. R. China

[b] Chemistry Department, College of Science, King Saud University, P.O. Box 2455, Riyadh 11451, Saudi Arabia.

[c] Theory of Nanomaterials Group, Department of Chemistry KU Leuven, Celestijnenlaan 200F, 3001 Leuven, Belgium. E-mail: Liviu.Chibotaru@kuleuven.be

[d] Department of Chemistry, School of Life Sciences, University of Sussex, Brighton, BN1 9QR, UK. Email: r.layfield@sussex.ac.uk

[e] Institute of Fundamental and Frontier Sciences, University of Electronic Science and Technology of China, Xiyuan Avenue 2006, Chengdu 611731, P. R. China.

Contents

Synthesis and Analytical Details 1-4
Single-Crystal X-ray Diffraction 5
Magnetic Measurements 6-14
Computational Details 15-20
References 21

Synthesis and Analytical Details

General Considerations

All reactions were carried out under rigorous anaerobic, anhydrous conditions under argon or nitrogen atmospheres using standard Schlenk-line and glovebox techniques. All solvents were refluxed over an appropriate drying agent for a minimum of three days (potassium for toluene and benzene, Na/K alloy for ether and hexane, CaH_2 for CH_2Cl_2) before distillation and were stored in ampoules over activated 4 Å molecular sieves. A literature procedure was used to synthesize $[(Cp^{Me})_2Dy(\mu\text{-}^nBu)]_2$.[1] 1-Naphthylthiol (NaphSH) was purchased from Sigma-Aldrich and was dried under a dynamic vacuum (10^{-3} mmHg) for several hours prior to use. Elemental analyses were carried out at London Metropolitan University, U.K. Powder XRD patterns were collected on a Malvern Panalytical X'Pert X-Ray Diffractometer.

Synthesis of $[(Cp^{Me})_2Dy(\mu\text{-SNaph})]_2\cdot(0.5\ Et_2O)$ ($Dy_2S_2\cdot(0.5\ Et_2O)$). At –40°C, a solution of 1-naphthylthiol (32 mg, 0.2 mmol) in Et_2O (1 ml) was slowly added to $[(Cp^{Me})_2Dy(\mu\text{-}^nBu)]_2$ (76 mg, 0.1 mmol) in Et_2O (1 ml), giving a colourless solution after warming to room temperature and stirring for one minute. The solution was then left undisturbed at room temperature overnight, resulting in the formation of colourless block-like crystals of **Dy_2S_2**$\cdot(0.5\ Et_2O)$, which were washed with cold Et_2O and isolated (48 mg, 49%). Placing the compound under vacuum for a few hours results in removal of the lattice solvent. Calculated for $C_{44}H_{42}S_2Dy_2$: C 55.05, H 4.41. Found: C 55.42, H 4.75. IR spectrum (cm^{-1}): 3076w, 3048w, 2975w, 2922w, 2858w, 1584w, 1557w, 1498w,

1454w, 1371w, 1249w, 1193w, 1154w, 1133w, 1109w, 1031m, 970m, 932w, 827m, 803m, 761s, 663m, 620w, 646w.

Synthesis of $[(Cp^{Me})_2Dy(\mu\text{-SNaph})]_3\cdot(1.5\ Et_2O)$ ($Dy_3S_3\cdot(1.5\ Et_2O)$). At –40°C, a solution of 1-naphthylthiol (32 mg, 0.2 mmol) as dissolved in Et_2O (1 ml), was slowly added to $[(Cp^{Me})_2Dy(\mu\text{-}^nBu)]_2$ (76 mg, 0.1 mmol) in Et_2O (1 ml), to give a colourless solution that was warmed to room temperature, stirred for one minute and then stored at –40°C overnight. The resulting pale-yellow plate-like crystals of $\mathbf{Dy_3S_3}\cdot(1.5\ Et_2O)$ were washed with cold Et_2O and isolated (30 mg, 36%). Placing the compound under vacuum for a few hours results in removal of the lattice solvent. Calculated for $C_{66}H_{63}Dy_3S_3$: C 55.05, H 5.41. Found C 55.75, H 5.07. IR spectrum (cm^{-1}): 3046w, 2966w, 2923w, 2859w, 1585w, 1559w, 1499w, 1460w, 1376w, 1251w, 1198w, 1154w, 1132w, 1109w, 1031m, 970m, 930w, 840m, 800m, 761s, 662m, 619w, 648w. Phase purity was confirmed by powder X-ray diffraction.

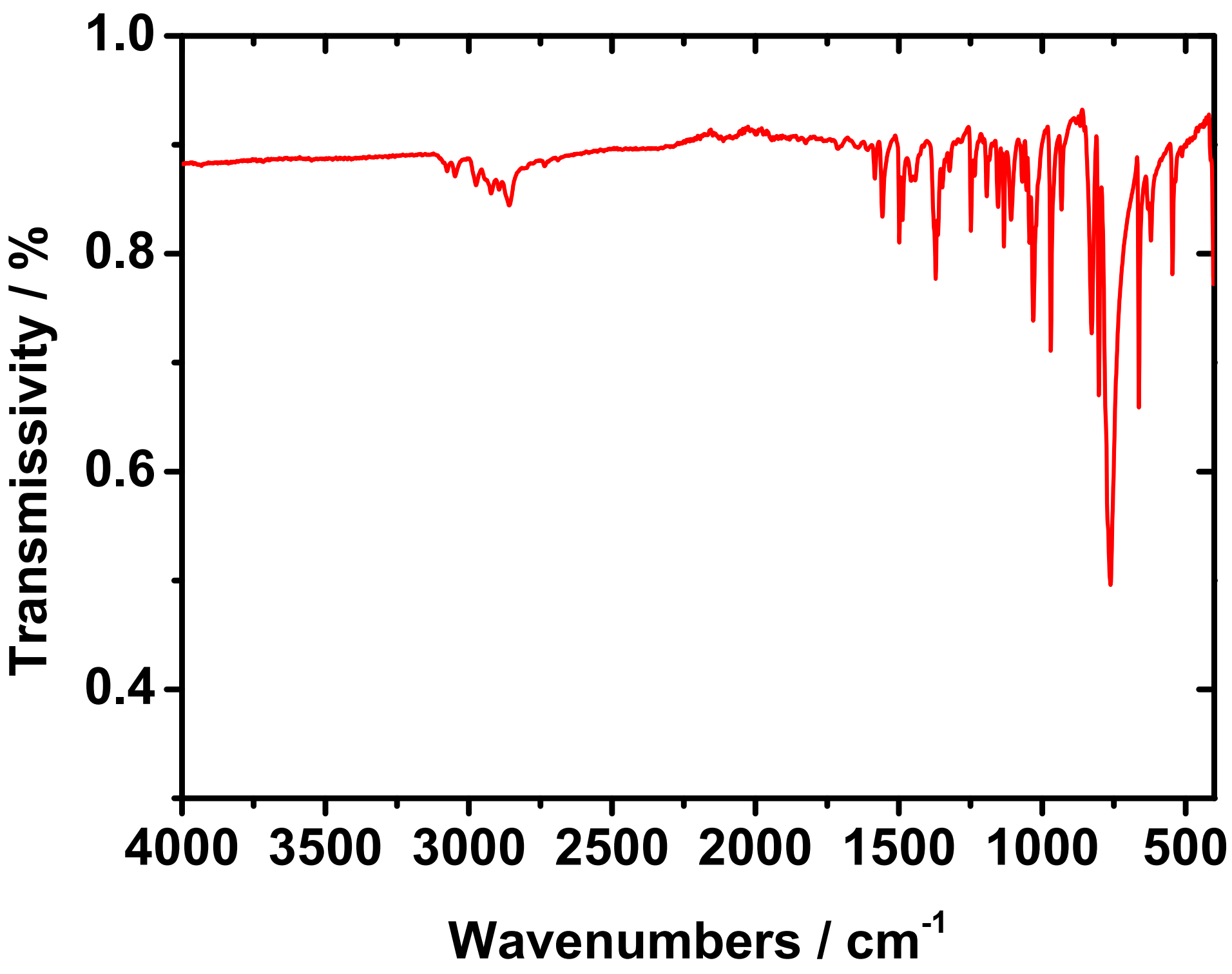

Figure S1. FTIR spectrum of solid $\mathbf{Dy_2S_2}$.

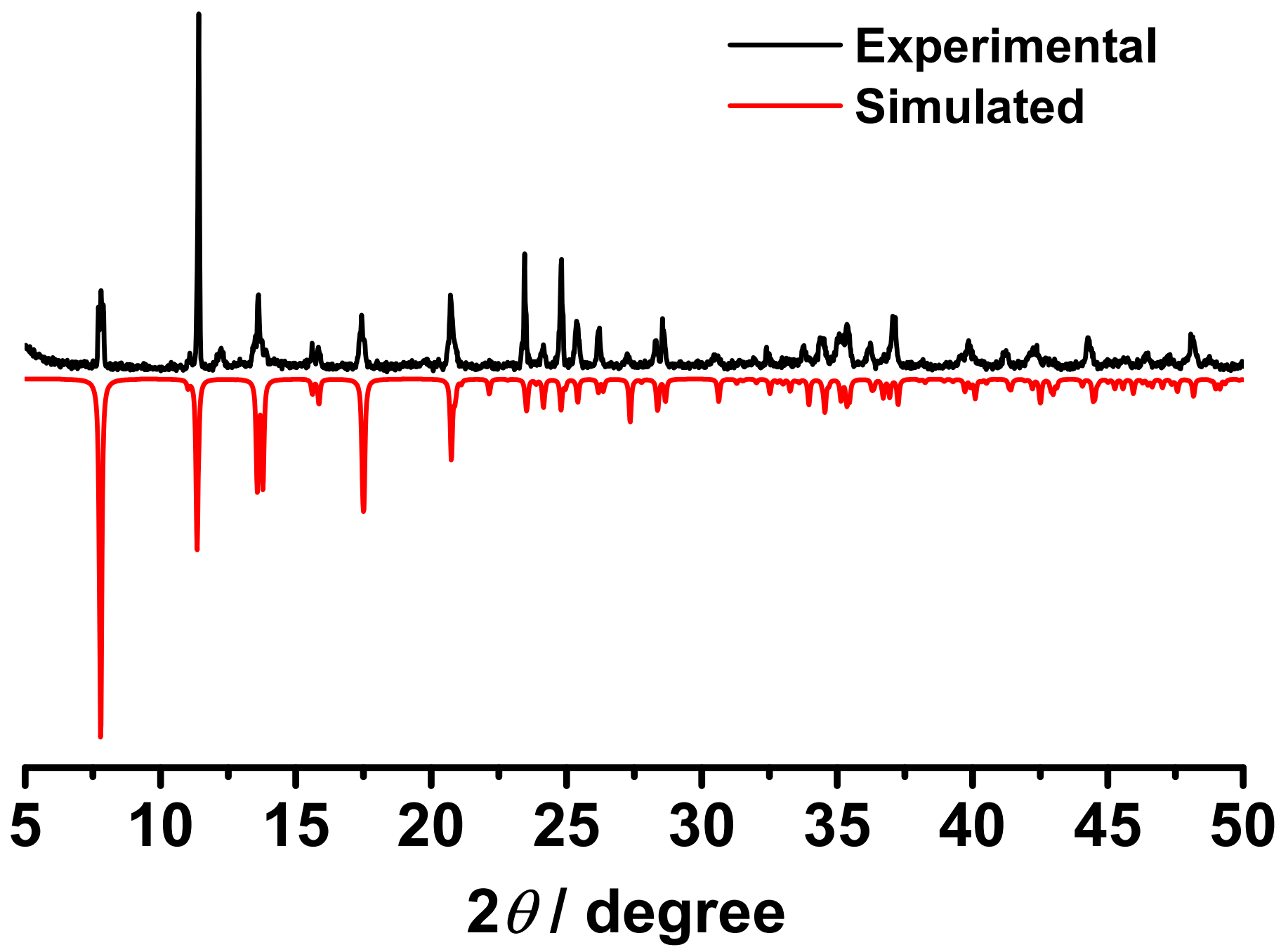

Figure S2. Powder XRD pattern of $\mathbf{Dy_2S_2}$.

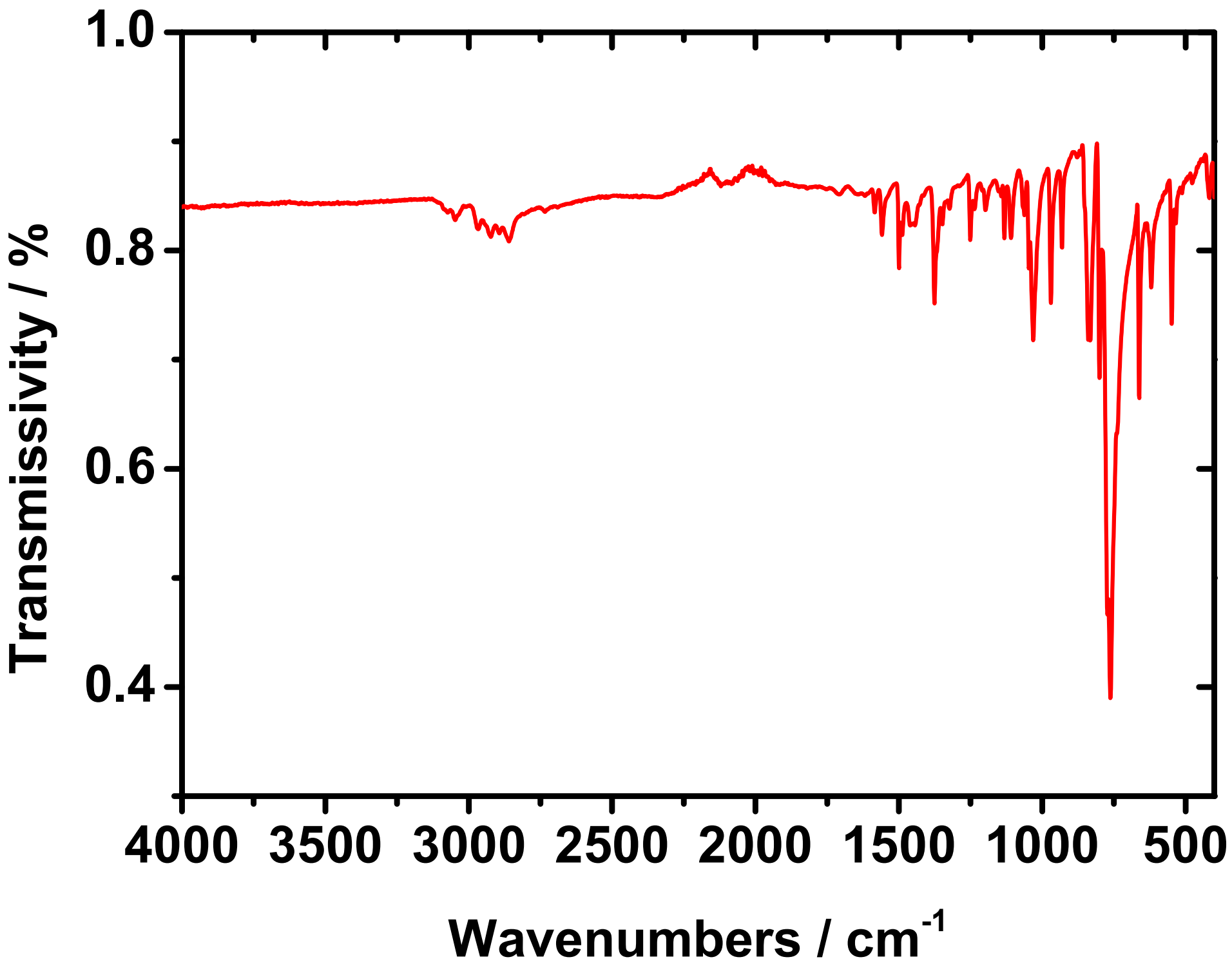

Figure S3. FTIR spectrum of solid $\mathbf{Dy_3S_3}$.

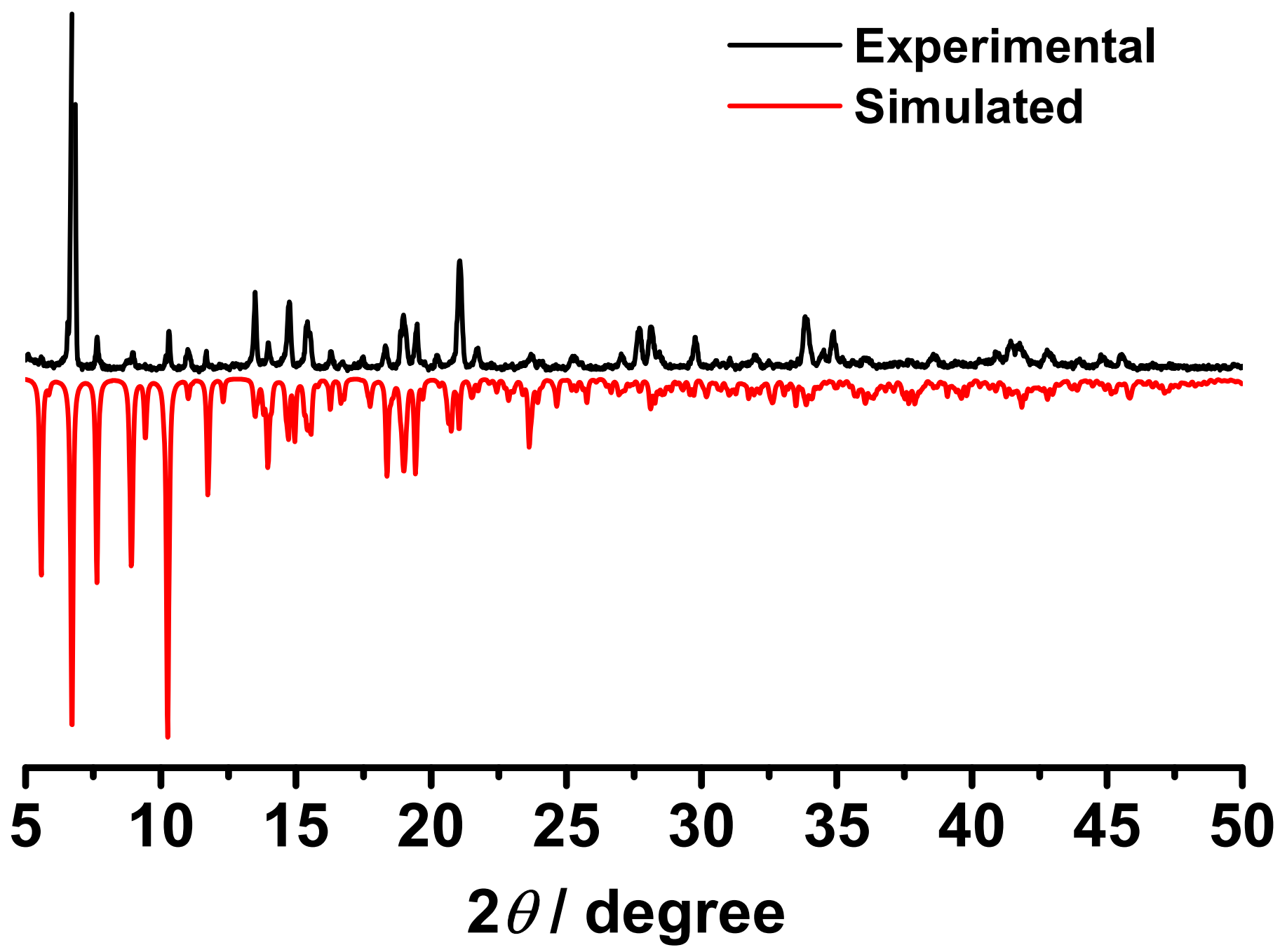

Figure S4. Powder XRD pattern of **Dy_3S_3**.

Single-Crystal X-ray Diffraction

X-ray diffraction data were collected on an Oxford Instruments XCalibur2 diffractometer or an Agilent SuperNova using Mo-K_α radiation. Data collection and processing (cell refinement, data reduction and absorption) of the two complexes were performed using *CrysAlisPro*. Structures were solved in Olex2 with SHELXT using intrinsic phasing and were refined with SHELXL using least squares minimization.[2-4] Anisotropic thermal parameters were used for the non-hydrogen atoms and isotropic parameters for the hydrogen atoms. Hydrogen atoms were added geometrically and refined using a riding model. Disordered solvent electron density was treated using the solvent-mask procedure implemented in Olex2.

Table S1. Crystal data and structure refinement for $\mathbf{Dy_2S_2}$ and $\mathbf{Dy_3S_3}$.

Name	$\mathbf{Dy_2S_2}$	$\mathbf{Dy_3S_3}$
CSD ref. code	2572223	2572224
Empirical formula	$C_{44}H_{42}Dy_2S_2$	$C_{66}H_{63}Dy_3S_3$
Formula weight	959.89	1439.84
Temperature / K	150	150
Crystal system	tetragonal	monoclinic
Space group	*I*4/m	*C*2/c
a/Å	16.0415(4)	35.247(3)
b/Å	16.0415(4)	19.8332(7)
c/Å	15.5697(7)	23.131(2)
α/°	90	90
β/°	90	131.682(15)
γ/°	90	90
Volume/Å^3	4006.6(3)	12077(3)
Z	4	8
ρ_{calc}g/cm^3	1.591	1.584
μ/mm^{-1}	3.833	3.815
F(000)	1880	5640
Crystal size/mm^3	0.1 × 0.07 × 0.05	0.09 × 0.08 × 0.02
Radiation	Mo Kα (λ = 0.71073)	Mo Kα (λ = 0.71073)
2Θ range for data collection/°	7.184 to 52.744	6.43 to 58.512
Index ranges	-18 ≤ h ≤ 20, -12 ≤ k ≤ 20, -10 ≤ l ≤ 19	-48 ≤ h ≤ 33, 0 ≤ k ≤ 25, 0 ≤ l ≤ 29
Reflections collected	5905	13913
Independent reflections	2116 [R_{int} = 0.0312, R_{sigma} = 0.0385]	13913 [R_{int} = 0.0301, R_{sigma} = 0.0512]
Data/restraints/parameters	2116/154/193	13913/81/631
Goodness-of-fit on F^2	1.06	1.066
Final R indexes [I>=2σ (I)]	R_1 = 0.0267, wR_2 = 0.0562	R_1 = 0.0525, wR_2 = 0.1237
Final R indexes [all data]	R_1 = 0.0336, wR_2 = 0.0592	R_1 = 0.0731, wR_2 = 0.1313
Largest diff. peak/hole / e Å^{-3}	0.69/-0.77	2.54/-1.67

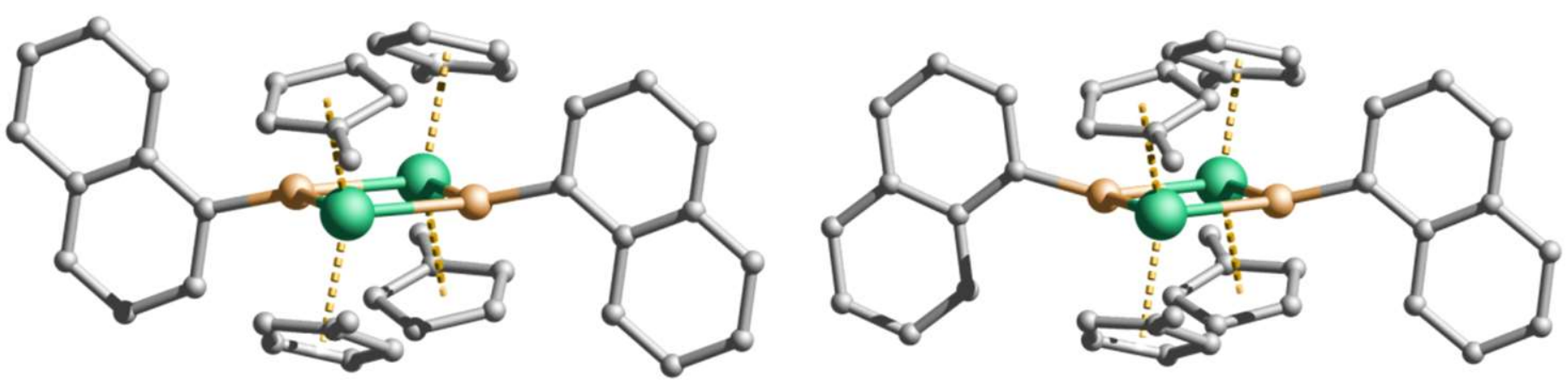

Figure S5. Structure of complex $\mathbf{Dy_2S_2}$ showing the two disordered components. Dy = green, C = grey, S = orange. Hydrogen atoms and solvent molecules have been omitted for clarity.

Magnetic Measurements

Finely ground microcrystalline powders of **Dy_2S_2** and **Dy_3S_3** were immobilized in eicosane inside an NMR tube and flame-sealed. Magnetization data were recorded on a Quantum Design MPMS-XL SQUID magnetometer equipped with a 7 T magnet. Variable-temperature magnetic susceptibility was measured in a DC field of 10000 Oe in the temperature range 2-300 K, and the frequency dependent AC susceptibility was measured with an oscillating field of 1.55 Oe and zero DC field. Diamagnetic corrections were applied using Pascal's constants.[5]

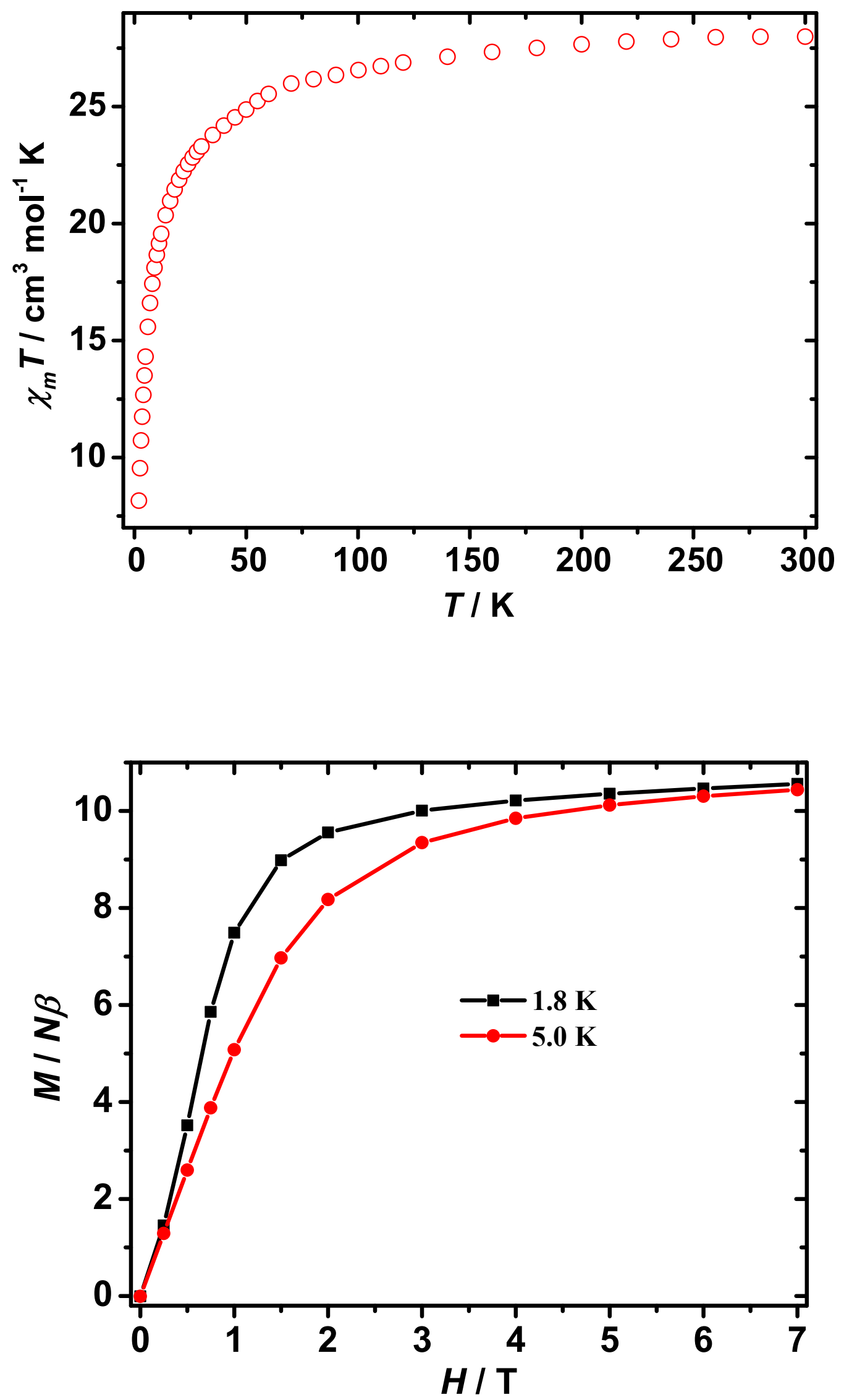

Figure S6. Upper: $\chi_M T$ versus T for **Dy_2S_2** collected at 1 T. Lower: Field dependence of the magnetization, M, at 1.8 and 5.0 K.

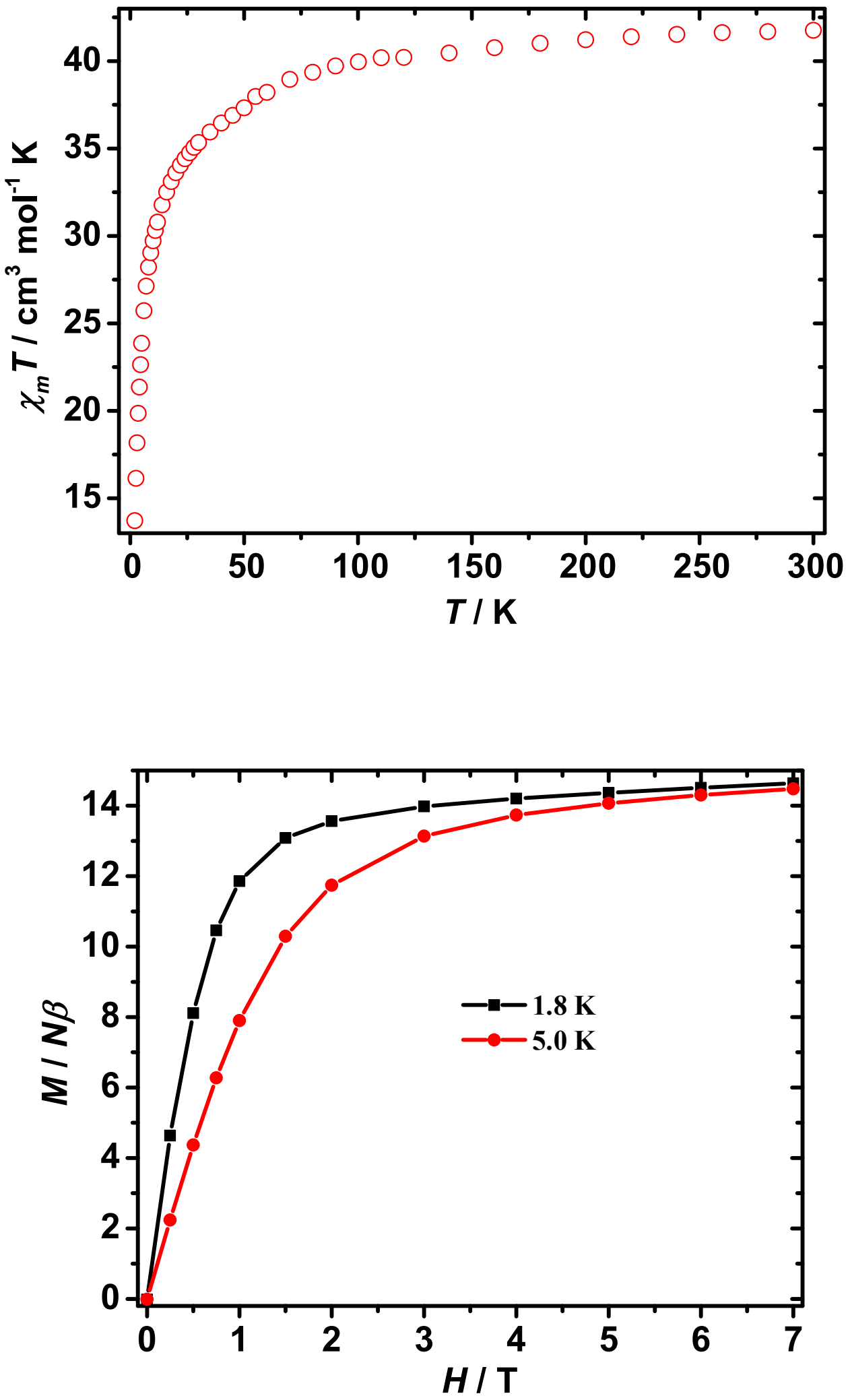

Figure S7. Upper: $\chi_M T$ versus T for **Dy_3S_3** collected at 1 T. Lower: Field dependence of the magnetization, *M*, at 1.8 and 5.0 K.

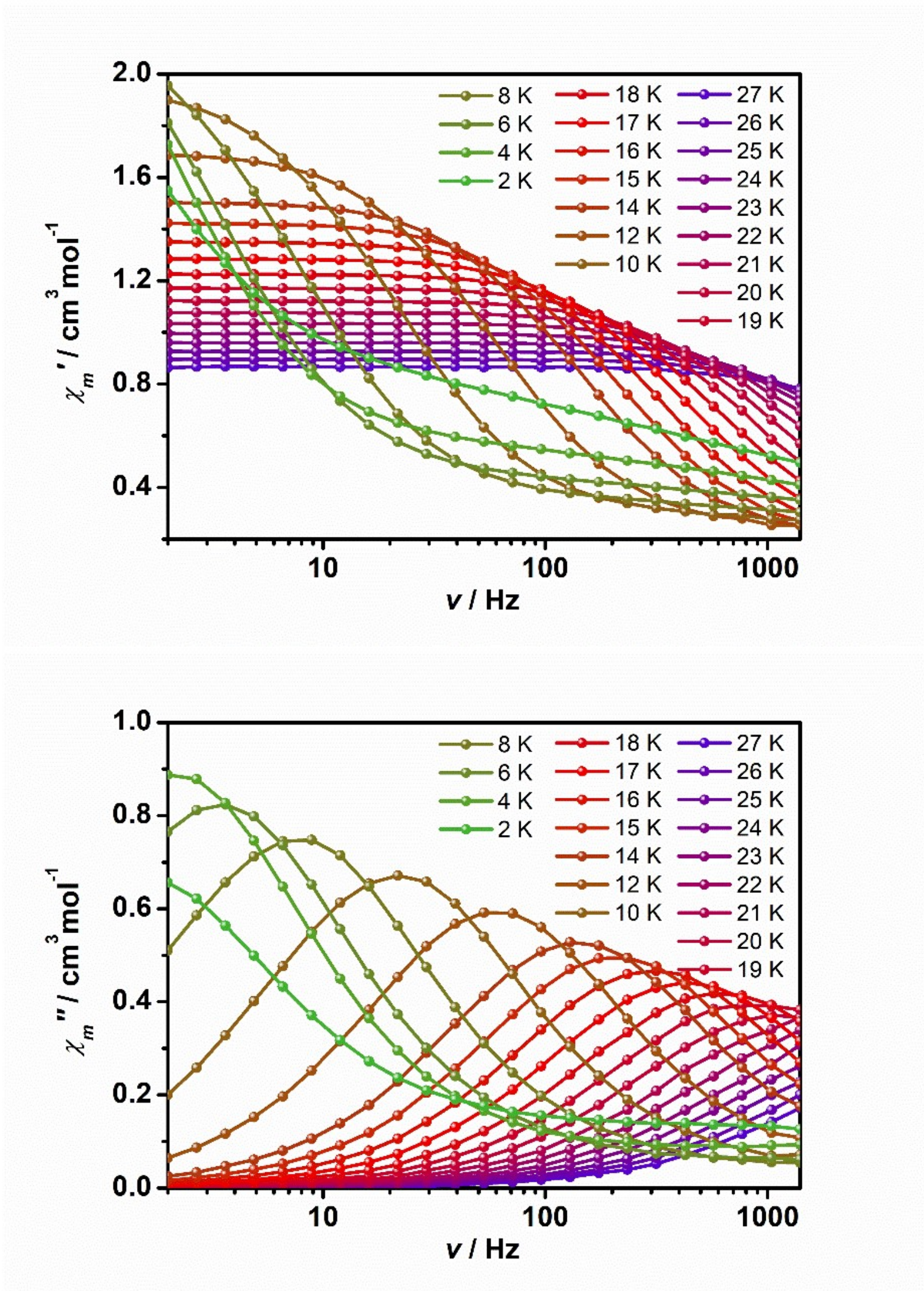

Figure S8. Plots of $\chi'(\nu)$ (upper) and $\chi''(\nu)$ (lower) for $\mathbf{Dy_2S_2}$ in zero applied field at the temperatures indicated. Solid lines represent best fits of the data using the parameters stated in Figure S10 and Table S2.

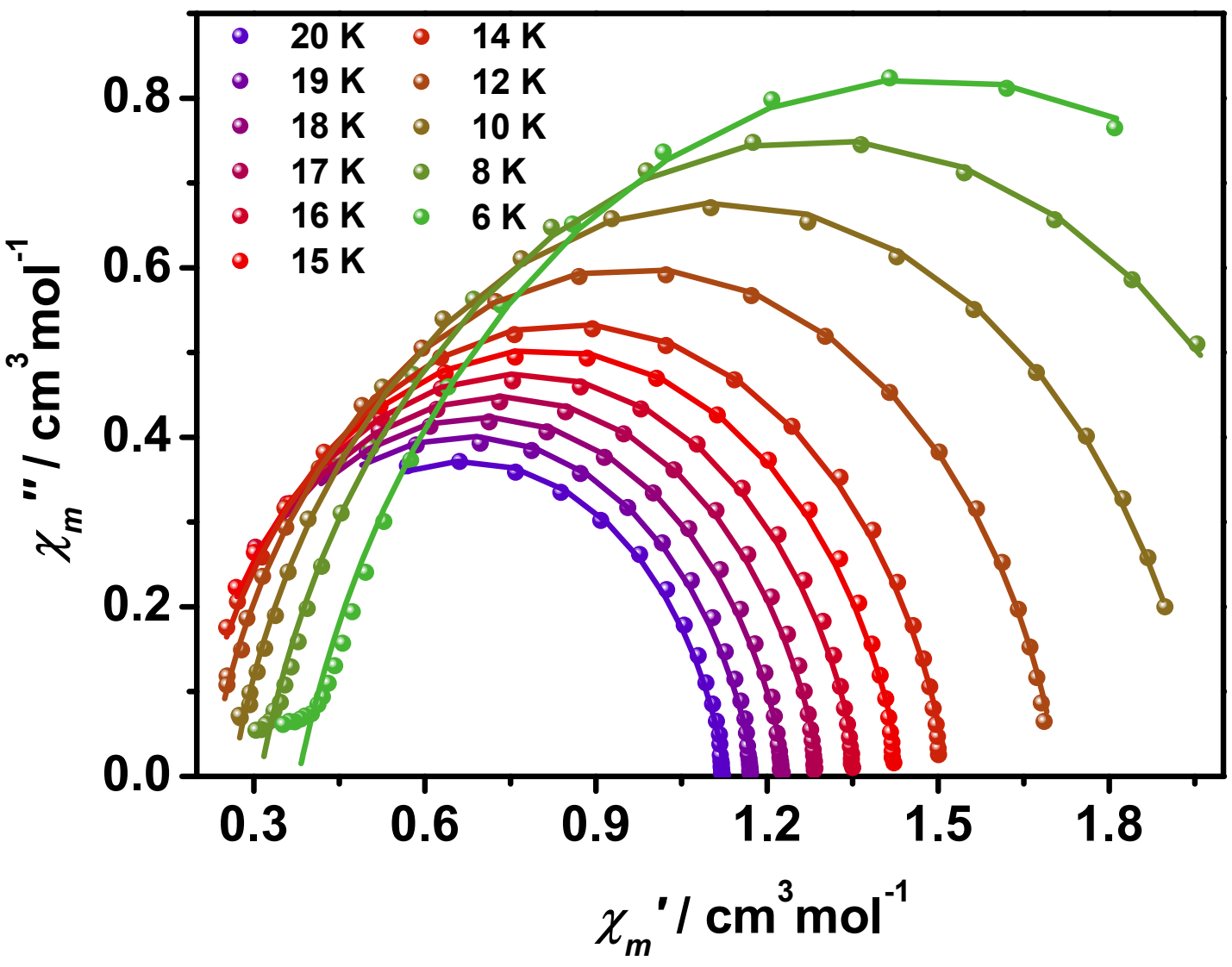

Figure S9. Cole-Cole plot using the AC susceptibility data for **Dy_2S_2**. The solid lines are the best fit obtained with a generalized Debye model.

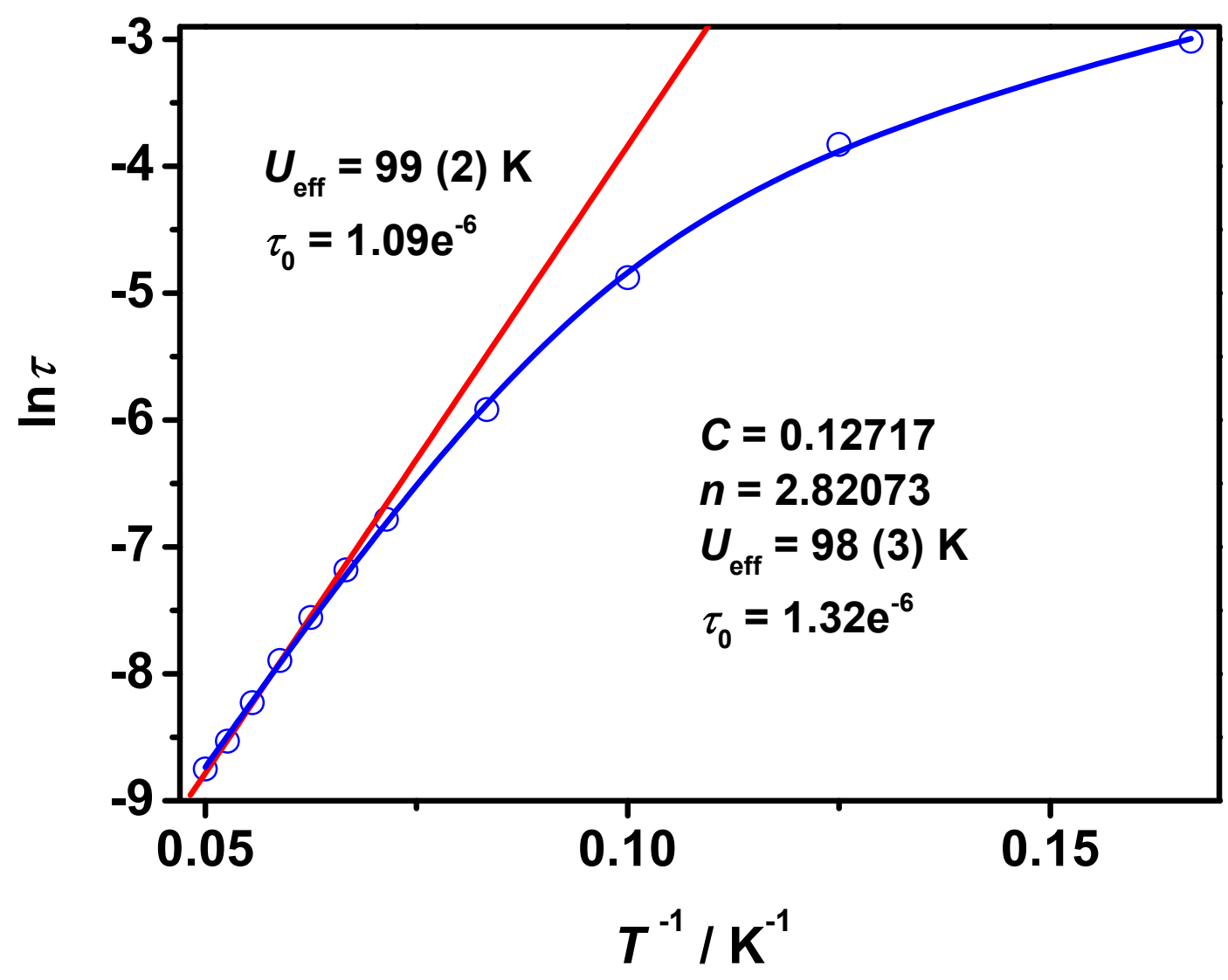

Figure S10. Plot of ln(τ) vs T^{-1}) for **Dy_2S_2**. The red line represents the best fit to the Arrhenius law of the thermally activated region, and the blue line is the best fits to the power law.

Table S2. Relaxation fitting parameters from least-squares fitting of the χ(ν) data for $\mathbf{Dy_2S_2}$.

T / K	τ/ s	α
20	1.58595E-4	0.13076
19	1.97326E-4	0.14416
18	2.67151E-4	0.14376
17	3.72177E-4	0.14172
16	5.2296E-4	0.14135
15	7.6101E-4	0.13836
14	0.00113	0.13548
12	0.00269	0.13648
10	0.00762	0.14712
8	0.02176	0.16604
6	0.04909	0.18588

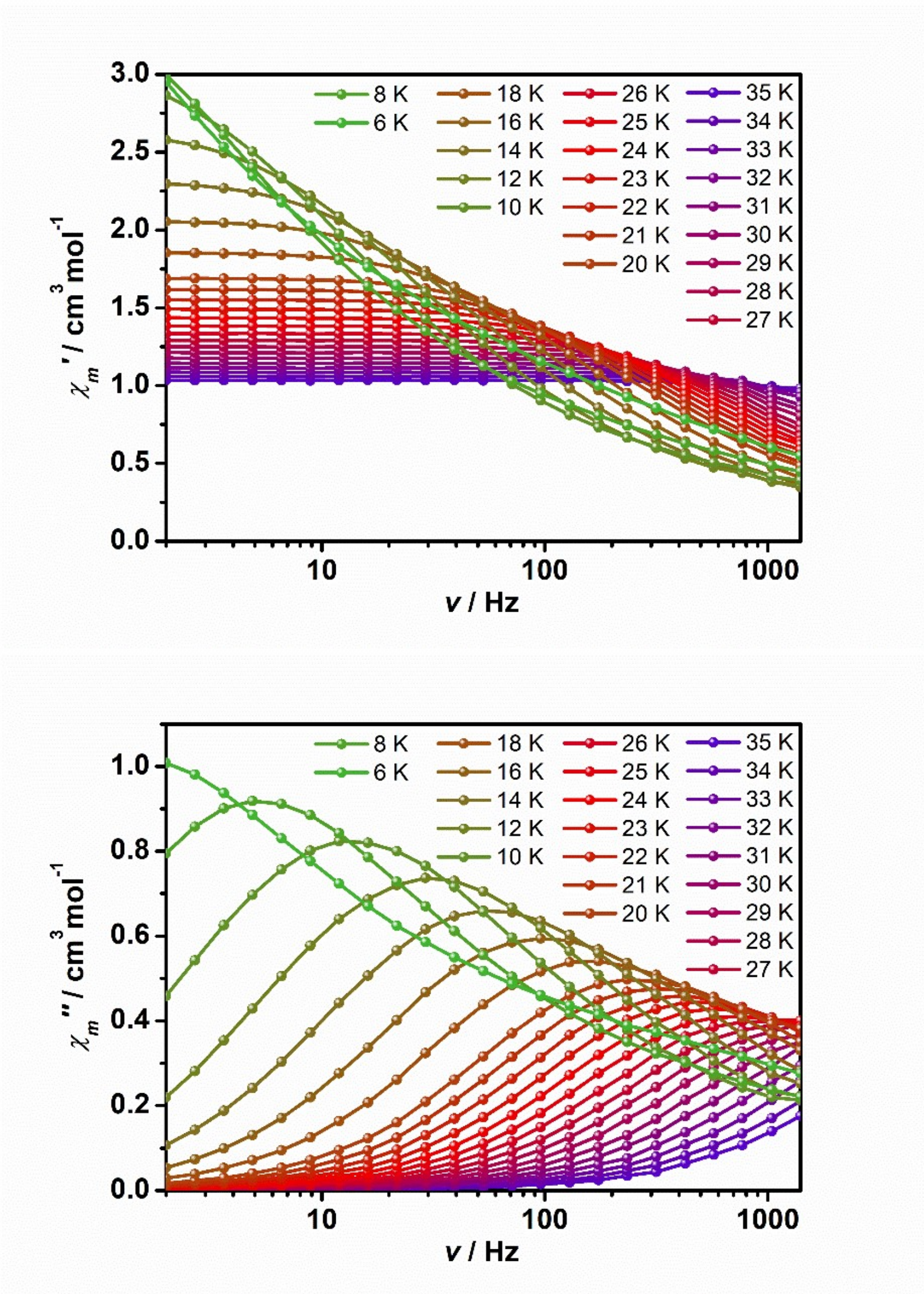

Figure S11. Plots of $\chi'(\nu)$ (upper) and $\chi''(\nu)$ (lower) for $\mathbf{Dy_3S_3}$ in zero applied field at the temperatures indicated. Solid lines represent best fits of the data using the parameters stated in Figure S13 and Table S3.

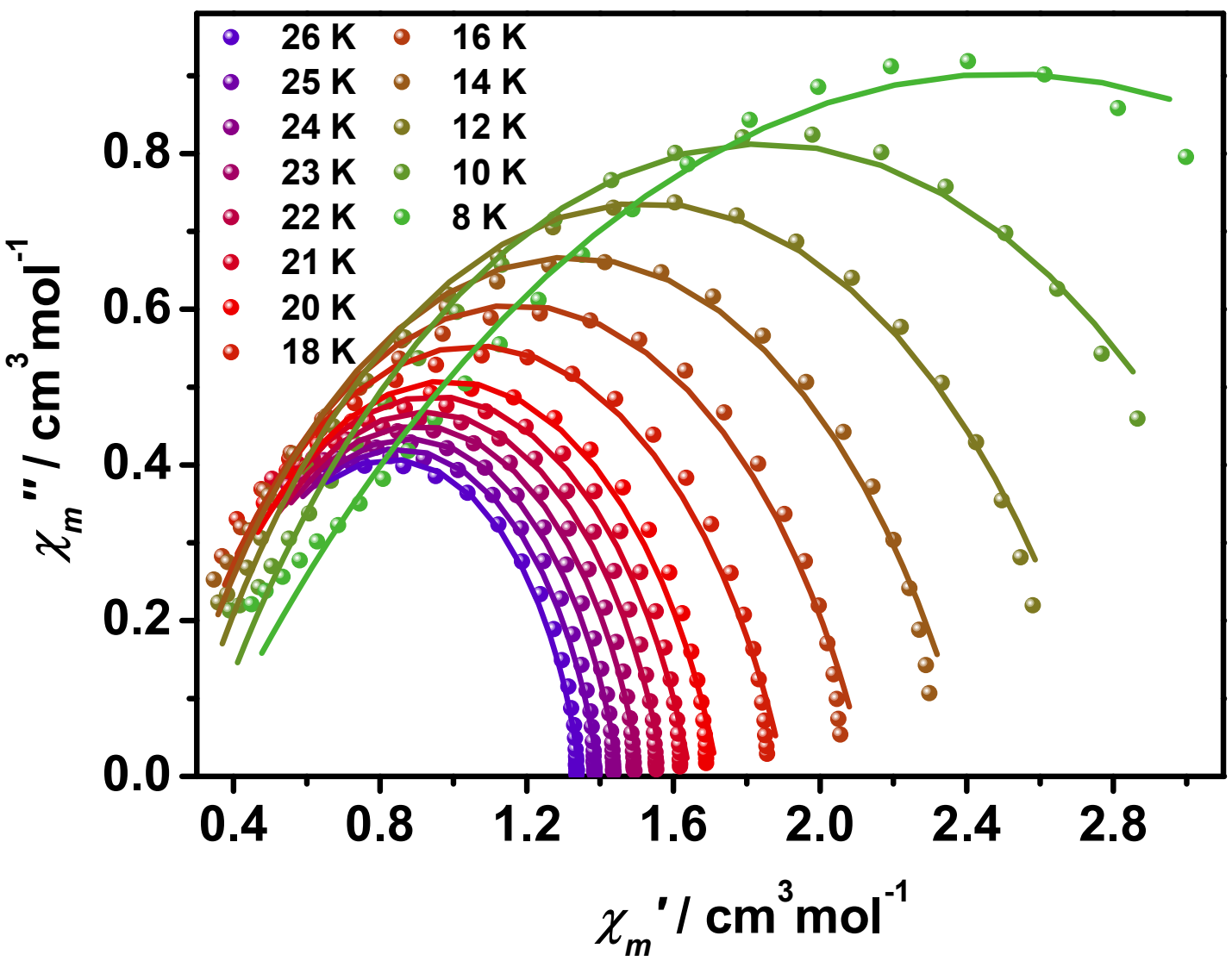

Figure S12. Cole-Cole plot using the AC susceptibility data for $\mathbf{Dy_3S_3}$. The solid lines are the best fit obtained with a generalized Debye model.

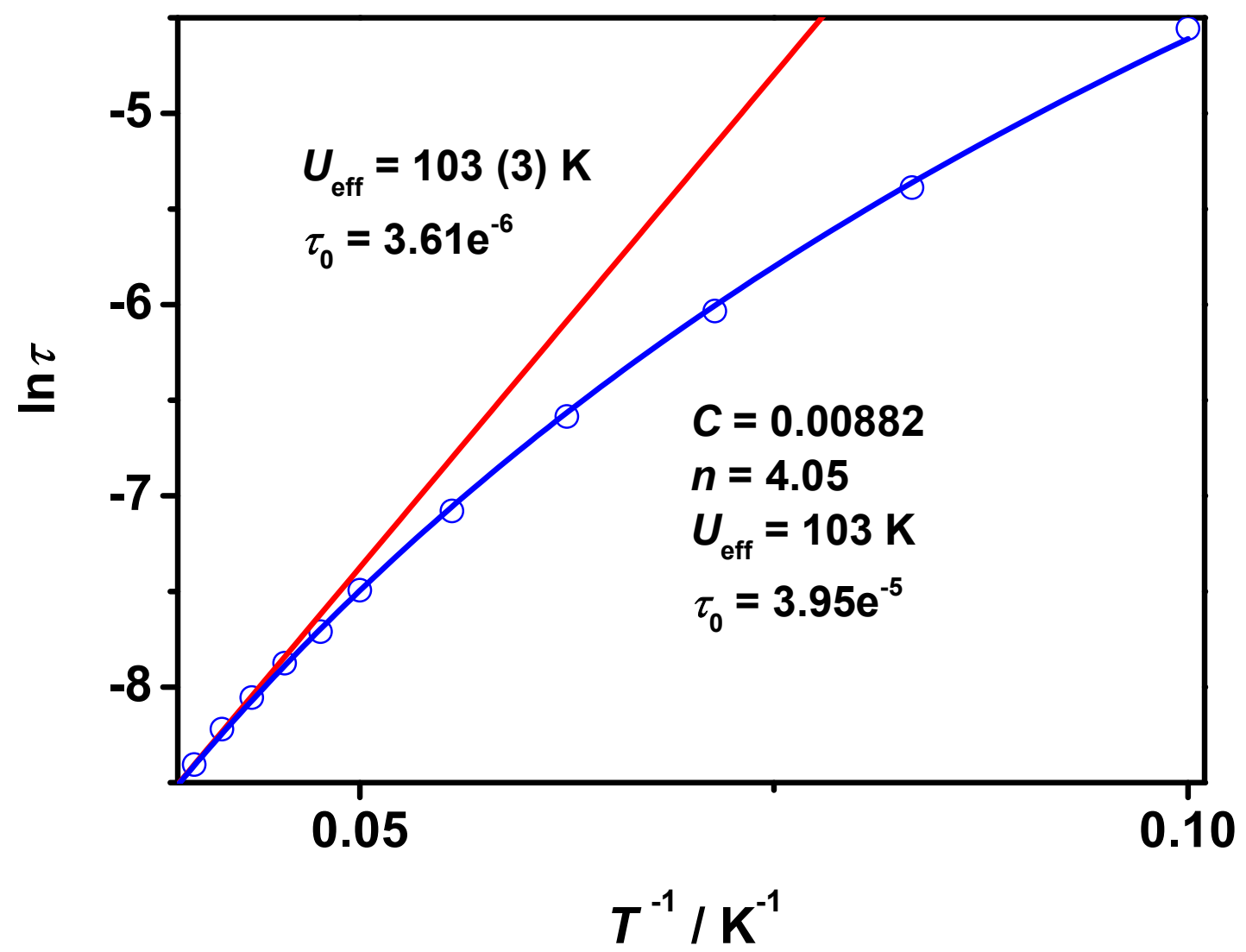

Figure S13. Plot of ln(t) vs T^{-1}) for $\mathbf{Dy_3S_3}$. The red line represents the best fit to the Arrhenius law of the thermally activated region, and the blue line is the best fits to the power law. (Note: the value of U_{eff} is fixed at 103 K (64 cm^{-1}), as obtained from the Arrhenius fitting results).

Table S3. Relaxation fitting parameters from Least-Squares Fitting of the $\chi(\nu)$ data for **Dy_3S_3**.

Temperature / K	τ / s	α
26	1.89839E-4	0.14897
25	2.23642E-4	0.16465
24	2.68989E-4	0.17464
23	3.17362E-4	0.19092
22	3.79721E-4	0.20495
21	4.47854E-4	0.22431
20	5.56722E-4	0.23205
18	8.42649E-4	0.26172
16	0.00138	0.2816
14	0.0024	0.30456
12	0.00457	0.33027
10	0.0105	0.3844
8	0.03878	0.50402

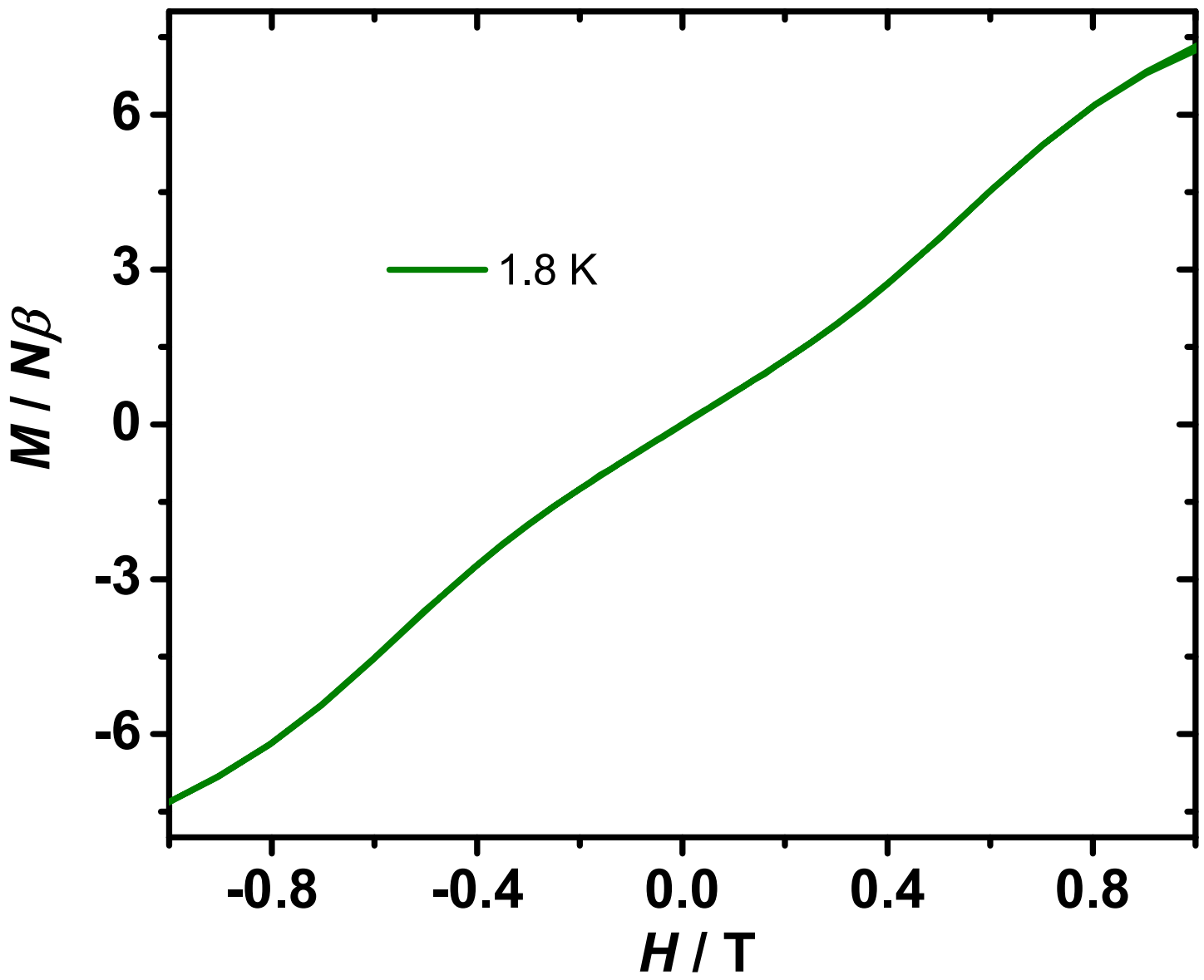

Figure S14. Hysteresis for **Dy_2S_2** at 1.8 K using field sweep rates of 2.5 mT s^{-1} for $|H|$ = 1-0.5 T, 1.4 mT s^{-1} for $|H|$ = 0.5-0.2 T, 0.75 mT s^{-1} for $|H|$ = 0.2-0.06 T, and 0.40 mT s^{-1} for $|H|$ = $\pm$0.06 T.

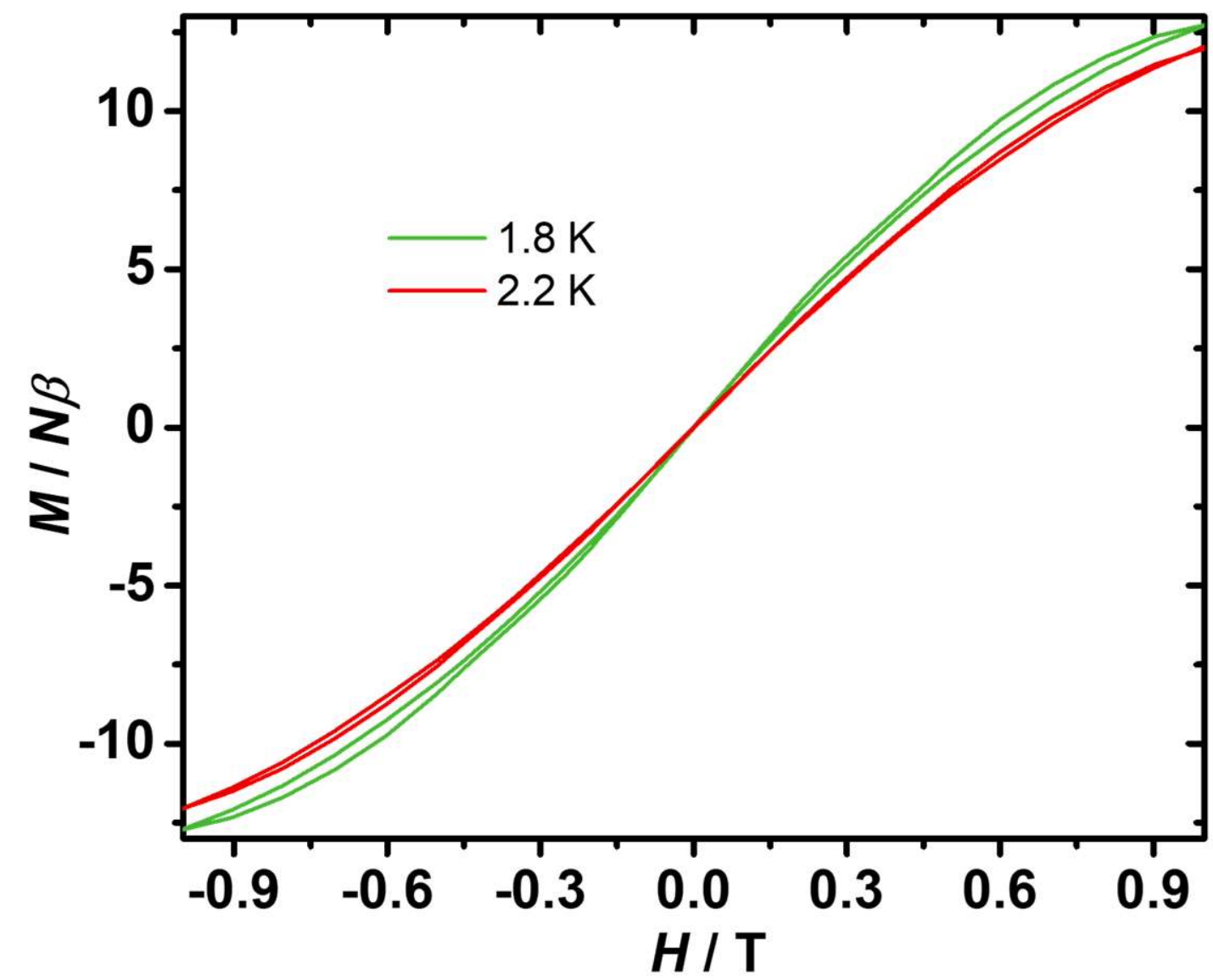

Figure S15. Hysteresis for **Dy_3S_3** at 1.8 K and 2.2 K using field sweep rates of 2.5 mT s^{-1} for $|H|$ = 1-0.5 T, 1.4 mT s^{-1} for $|H|$ = 0.5-0.2 T, 0.75 mT s^{-1} for $|H|$ = 0.2-0.06 T, and 0.40 mT s^{-1} for $|H|$ = $\pm$0.06 T.

Computational Details

Structures and the main magnetic axes of the ground Kramer doublet for $\mathbf{Dy_2S_2}$ and $\mathbf{Dy_3S_3}$ are shown in Figure 1 and Figure 2. Two basis set approximations have been employed: basis set 1 (small) and basis set 2 (large). Table S4 shows the contractions of the employed basis sets for all elements in the complexes.

Table S4. Contractions of the employed basis sets in computational approximations.

Basis 1	Basis 2
Dy.ANO-RCC...VDZP.	Dy.ANO-RCC... VTZP.
Lu.ANO-RCC...VDZ.	Lu.ANO-RCC... VDZ.
S.ANO-RCC... VDZ.	S.ANO-RCC... VDZP.
C.ANO-RCC... VDZ.	C.ANO-RCC... VDZP.
H.ANO-RCC... VDZ.	C.ANO-RCC... VDZ.
	H.ANO-RCC... VDZ.

Table S5. *Ab initio* results for the *J*= 15/2 multiplet of Dy1 in complex $\mathbf{Dy_2S_2}$.

KDs	CASSCF
	Crystal field wavefunction
1	90.5%|±15/2⟩+9.2%|±11/2⟩
2	93.7%|±13/2⟩
3	85%|±11/2⟩+9.2%|±15/2⟩
4	62%|±9/2⟩+13.8%|±1/2⟩+8%|±5/2⟩
5	27%|±9/2⟩+10.3%|±5/2⟩+26.7%|±3/2⟩+30.6%|±1/2⟩
6	67.6%|±7/2⟩+9.3%|±3/2⟩+12.2%|±1/2⟩
7	55.9%|±5/2⟩+16.6%|±7/2⟩+23.8%|±3/2⟩
8	41.8%|±1/2⟩+33.4%|±3/2⟩+18.4%|±5/2⟩

Table S6. *Ab initio* results for the *J*= 15/2 multiplet of Dy1 in complex $\mathbf{Dy_3S_3}$.

KDs	CASSCF
	Crystal filed wavefunction
1	89.7%|±15/2⟩+10.1%|±11/2⟩
2	93.4%|±13/2⟩
3	84.3%|±11/2⟩+9.9%|±15/2⟩
4	32.8%|±3/2⟩+44.3%|±1/2⟩+16.9%|±5/2⟩
5	85.2%|±9/2⟩+4.7%|±5/2⟩
6	15.8%|±5/2⟩+58.8%|±7/2⟩+11.6%|±3/2⟩
7	45%|±5/2⟩+28.2%|±7/2⟩+20.7%|±3/2⟩
8	43.8%|±1/2⟩+33.3%|±3/2⟩+17.4%|±5/2⟩

Table S7. *Ab initio* results for the *J*= 15/2 multiplet of Dy2 in complex $\mathbf{Dy_3S_3}$.

KDs	CASSCF
	**Crystal field wavefunction**
1	91.5%|±15/2⟩+8.4%|±11/2⟩
2	96.6%|±13/2⟩
3	81.5%|±11/2⟩+7.9%|±15/2⟩
4	64.6%|±9/2⟩+15.6%|±5/2⟩
5	40.9%|±9/2⟩+11.3%|±7/2⟩+37.5%|±1/2⟩
6	65.4%|±7/2⟩+13.2%|±5/2⟩+8.5%|±3/2⟩
7	15.6%|±9/2⟩+15.2%|±7/2⟩+52.4%|±5/2⟩+11.4%|±3/2⟩
8	44.1%|±1/2⟩+38.1%|±3/2⟩+17%|±5/2⟩

Table S8. *Ab initio* results for the *J*= 15/2 multiplet of Dy3 in complex $\mathbf{Dy_3S_3}$.

KDs	CASSCF
	**Crystal field wavefunction**
1	92.3%|±15/2⟩+7.3%|±11/2⟩
2	93.6%|±13/2⟩
3	85.2%|±11/2⟩
4	34%|±9/2⟩+11.6%|±5/2⟩+16.7%|±3/2⟩+26.1%|±1/2⟩
5	56.1%|±9/2⟩+14.3%|±3/2⟩+13%|±1/2⟩
6	68.1%|±7/2⟩+14.8%|±1/2⟩+9.8%|±3/2⟩
7	56.6%|±5/2⟩+24.7%|±3/2⟩+15.4%|±7/2⟩
8	43.8%|±1/2⟩+34.3%|±3/2⟩+17.3%|±5/2⟩

Table S9. The angles between the orientations of the magnetization in the excited KDs relative to the ground KD for all Dy^{3+} centres **Dy_2S_2** and **Dy_3S_3**.

Compound	Center	Excited relative to ground	Angle(°)
**Dy_2**	Dy	first	7.6
		second	3.1
		third	32.6
		fourth	73.2
		fifth	74.5
		sixth	88.1
		seventh	91.2
**Dy_3**	Dy1	first	5.8
		second	3.8
		third	84.8
		fourth	5.9
		fifth	90.2
		sixth	91.4
		seventh	90.4
	Dy14	first	5.1
		second	12.1
		third	2.5
		fourth	6.5
		fifth	60.4
		sixth	113.2
		seventh	90.9
	Dy16	first	8.6
		second	5.8
		third	68.3
		fourth	47.5
		fifth	66.8
		sixth	90.9
		seventh	91.2

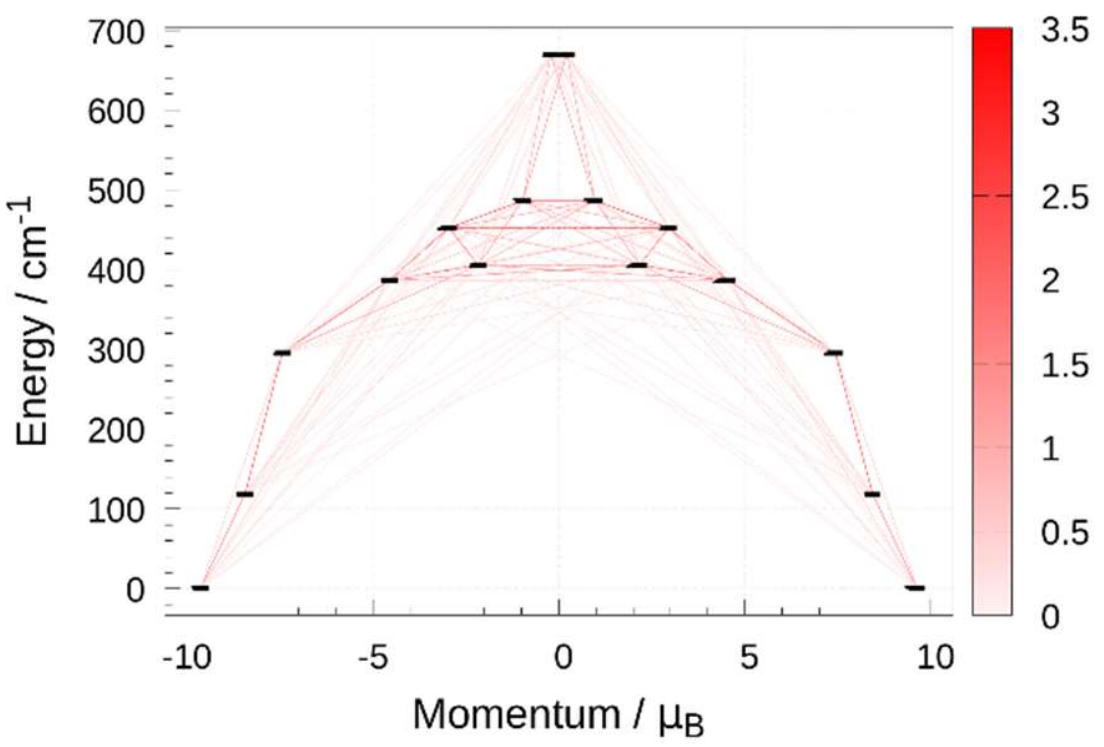

Figure S16. Calculated relaxation barrier for **Dy_2S_2**. The states are placed on the diagram according to their magnetic moments (bold black lines). The horizontal red lines show the tunneling transitions (the energy splitting) within each doublet state, while the non-horizontal lines show the spin-phonon transition paths. The numbers at non-horizontal lines are averaged transition moments in μ_B connecting the corresponding states. The numbers at horizontal lines are tunneling gaps. The green arrows delineate the relaxation path.

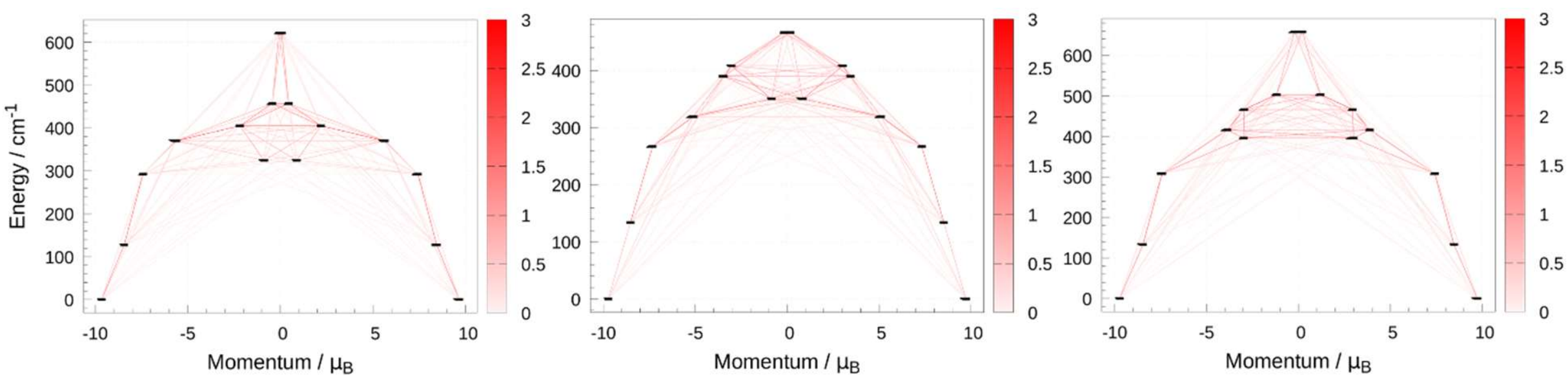

Figure S17. Calculated relaxation barrier for the three Dy^{3+} centres in **Dy_3S_3**.

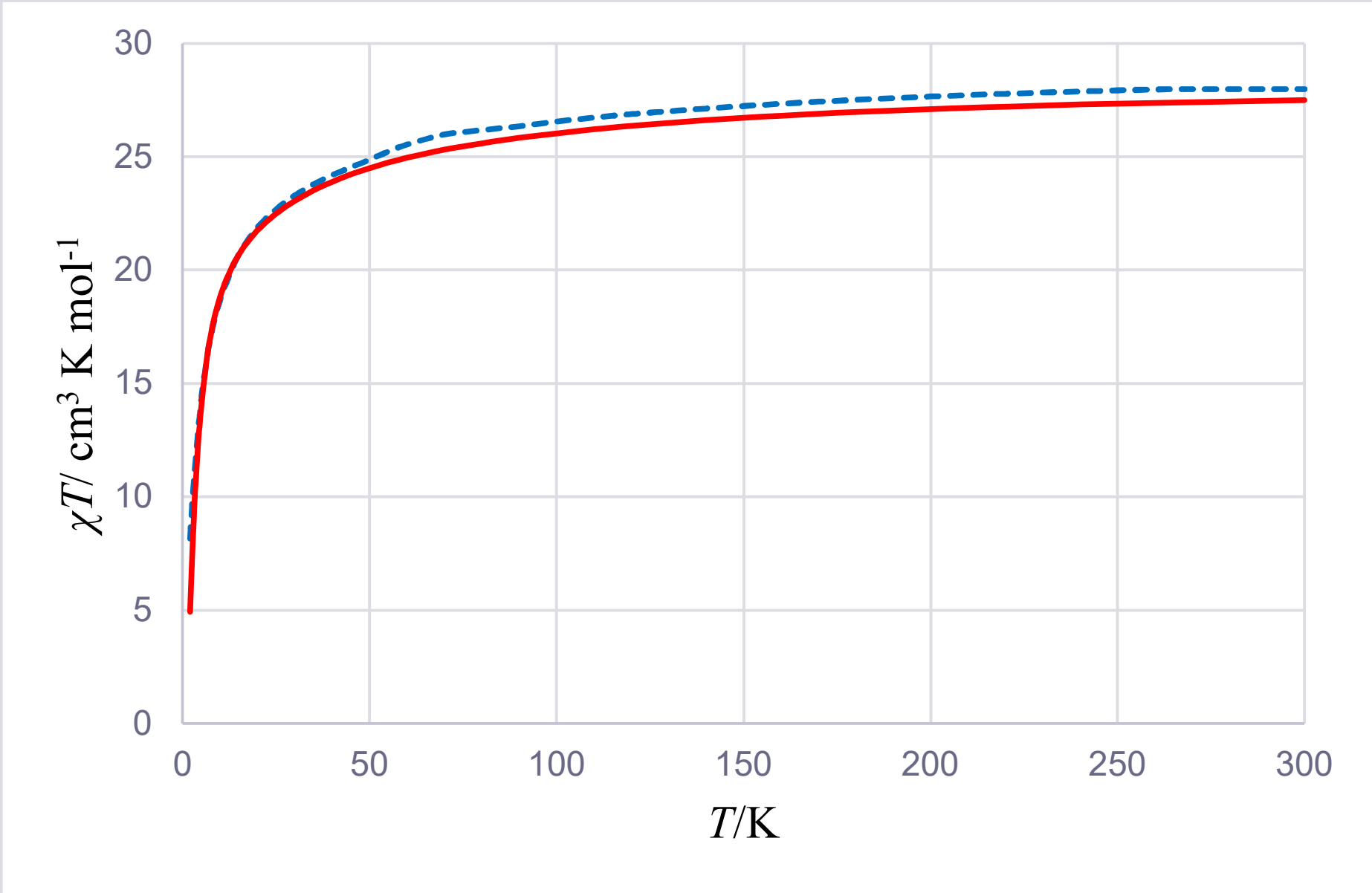

Figure S18. Experimental (dashed blue line) vs. calculated magnetic susceptibility data of $\mathbf{Dy_2S_2}$.

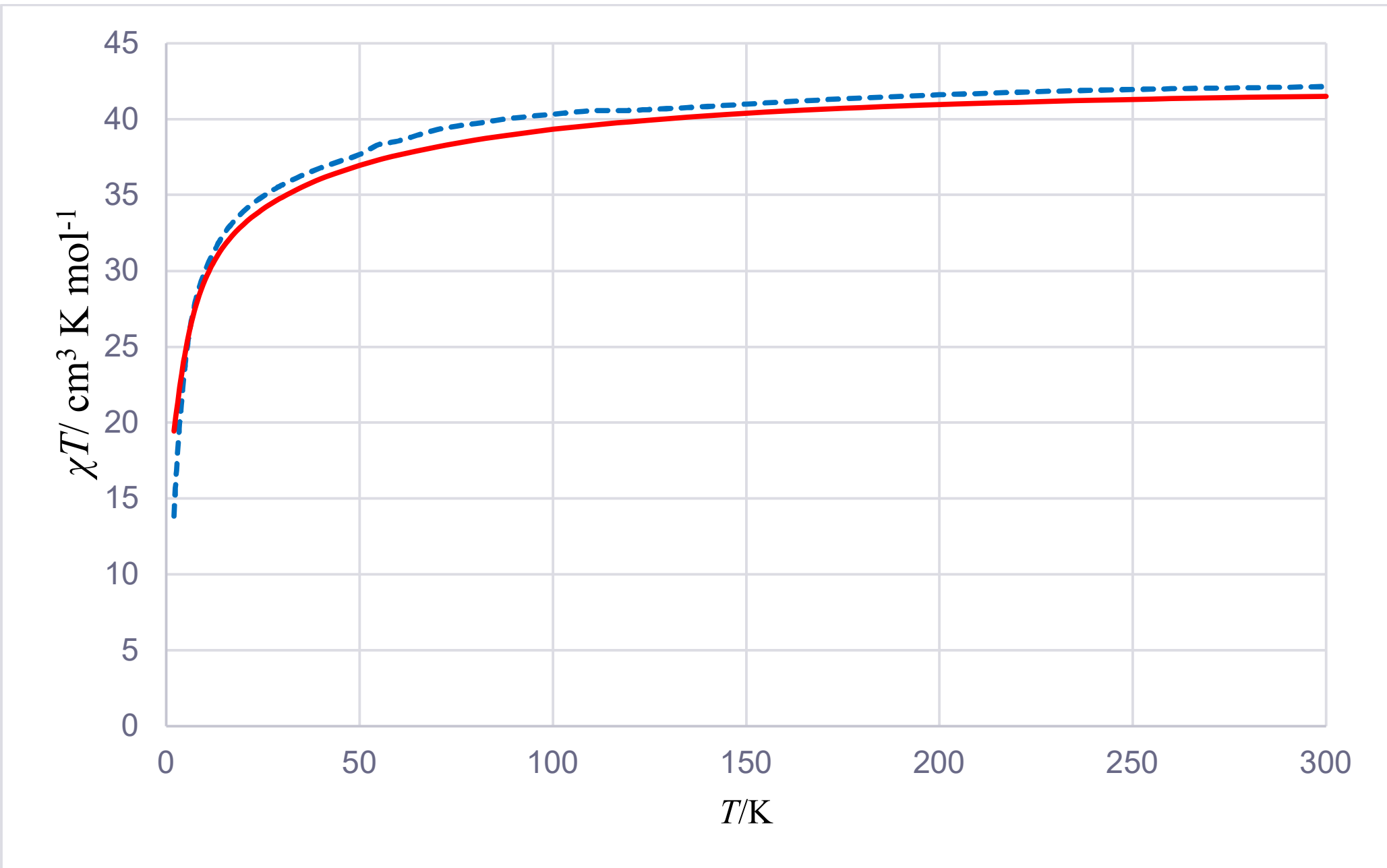

Figure S19. Experimental (dashed blue line) vs. calculated magnetic susceptibility data of $\mathbf{Dy_3S_3}$.

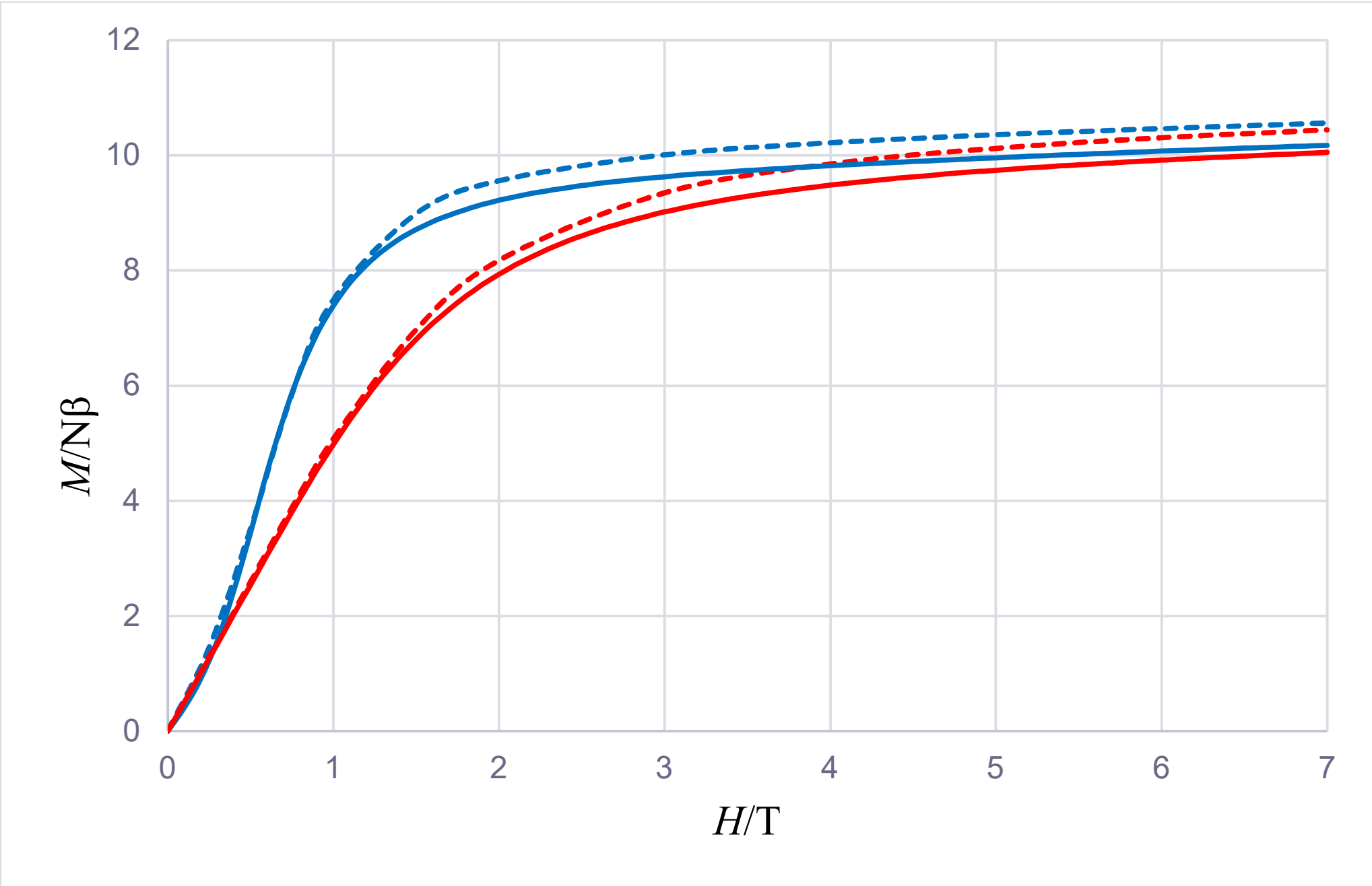

Figure S20. Experimental (dashed line) vs. calculated (solid line) magnetization data of **Dy_2S_2**. The blue and red lines correspond to the data at 1.8K and 5K, respectively.

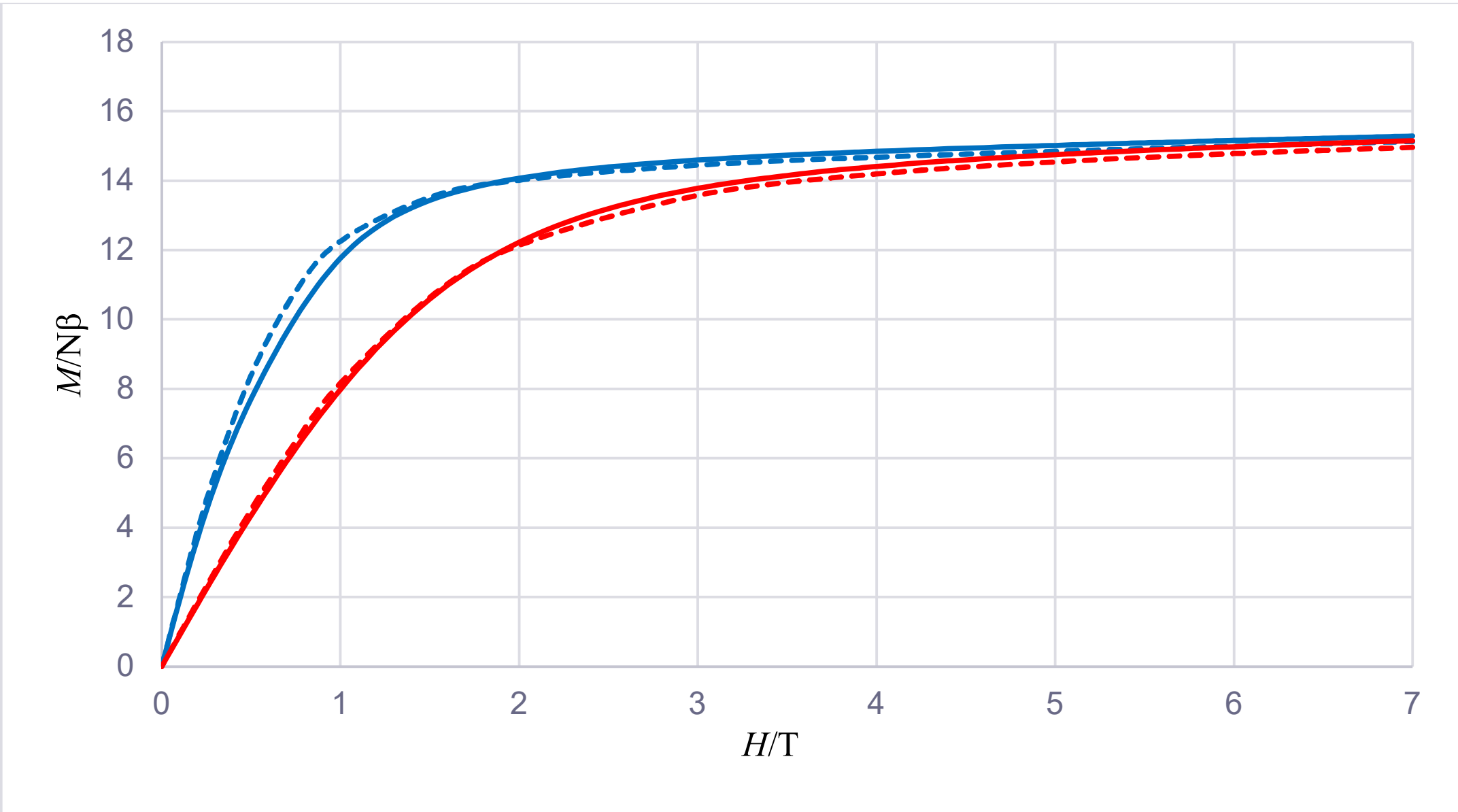

Figure S21. Experimental (dashed line) vs. calculated (solid line) magnetization data of **Dy_3S_3**. The blue and red lines correspond to the data at 1.8K and 5K, respectively.

References

1. R. Grindell, B. M. Day, F. S. Guo, T. Pugh and R. A. Layfield, Activation of C-H bonds by Rare-Earth Metallocene-Butyl Complexes, *Chem. Commun*, 2017, **53**, 9990-9993.
2. O. V. Dolomanov, L. J. Bourhis, R. J. Gildea, J. A. K. Howard, and H. Puschmann, OLEX2: A Complete Structure Solution, Refinement and Analysis Program. *J. Appl. Crystallogr*. 2009, **42**, 339-341.
3. L. J. Bourhis, O. V. Dolomanov, R. J. Gildea, J. A. K. Howard, and H. Puschmann, The Anatomy of a Comprehensive Constrained, Restrained Refinement Program for the Modern Computing Environment – Olex2 Dissected. *Acta Crystallogr. A* 2015, **71**, 59-75.
4. G. M. Sheldrick, Crystal Structure Refinement with SHELXL. *Acta Crystallogr. C* 2015, **71**, 3-8.
5. G. A. Bain, J. F. Berry, Diamagnetic Corrections and Pascal's Constants. *J. Chem. Ed.* 2008, **85**, 532-532
